# Neural-enhancing PRP/Alg/GelMA triple-network hydrogel for neurogenesis and angiogenesis after spinal cord injury via PI3K/AKT/mTOR signaling pathway

**DOI:** 10.7150/thno.109091

**Published:** 2025-03-03

**Authors:** Zhihe Yun, Jiuping Wu, Xinzhi Sun, Tao Yu, Wu Xue, Anyuan Dai, Tianyang Yuan, Xuhao Yang, Inbo Han, Yanting Liu, Wenlong Song, Qinyi Liu

**Affiliations:** 1 Department of Orthopedics, The Second Hospital of Jilin University, Changchun 130041, China.; 2 Department of Orthopedics, The First Affiliated Hospital of Zhengzhou University, Zhengzhou 450052, China.; 3 Center for Translational Medicine, The First Affiliated Hospital of Zhengzhou University, Zhengzhou 450052, China.; 4 State Key Laboratory of Supramolecular Structure and Materials, College of Chemistry, Jilin University, Changchun 130023, China.; 5 Department of Neurosurgery, CHA University School of Medicine, CHA Bundang Medical Center, 59 Yaptap-ro, Bundang-gu, Seongnam-si, Gyeonggi-do 13496, Republic of Korea.

**Keywords:** spinal cord injury, platelet-rich plasma, brain-derived neurotrophic factor, hydrogels, nerve regeneration, angiogenesis

## Abstract

**Rational:** Spinal cord injury (SCI) is among the most devastating conditions affecting the central nervous system. Nerve regeneration and vascular regeneration are two important strategies for SCI. While platelet-rich plasma (PRP) gel is a bio-hydrogel enriched with a large number of growth factors. It has an excellent ability to promote blood vessel regeneration, but its role in nerve repair needs to be further enhanced. In addition, its weak mechanical properties and rapid release of components have limited its efficacy.

**Method:** In this study, we cleverly applied the dual effects of activation and cross-linking of calcium ions to construct a novel PRP/ sodium alginate (Alg)/ gelatin methacrylate (GelMA) triple-networked hydrogel and enhanced the hydrogel's function in promoting nerve regeneration using brain-derived neurotrophic factor (BDNF).

**Results:** *In vitro* experiments, we verified the biomimetic, injectable, biodegradable, biocompatible, vascular regenerative and neural regenerative properties of the neural-enhancing triple-network hydrogel by involving endothelial cells and neural stem cells (NSCs). When implanted into SCI rats, this hydrogel significantly improved its motor function. It promotes neuronal differentiation, inhibits astrocyte differentiation, supports axonal regeneration, and enhances the migration of endogenous NSCs and vascular regeneration. In addition, this hydrogel may facilitate neurogenesis and angiogenesis via activating the PI3K/AKT/mTOR signaling pathway.

**Conclusions:** The neural regeneration-enhanced triple-network hydrogel we developed has excellent dual biological functions of neural regeneration and angiogenesis, and it is a straightforward, viable, and promising therapeutic strategy for SCI regeneration.

## Introduction

Traumatic spinal cord injury (SCI) is an acute neurodegenerative condition caused by traumatic events, leading to permanent motor impairment, sensory deficits, and autonomic dysfunction, placing a substantial burden on both patients and society 1, 2. The pathophysiology of SCI is highly complex, involving neural damage such as neuron loss, axonal degeneration, and myelin sheath necrosis, alongside microvascular damage, including cell death and tissue destruction resulting from vascular injury 3, 4. Nerve regeneration and vascular regeneration are two important strategies for SCI repair. The degree of nerve regeneration after SCI determines the recovery of function, and vascular regeneration can assist nerve regeneration by providing nutrition support. During the repair of SCI, axonal regeneration usually grows along blood vessels, and disruption of vascular regeneration prevents regeneration of injured tissue 5. Therefore, the close interaction between blood vessels and neurons plays a crucial role in maintaining and repairing the nervous system 6, 7.

Currently, platelet-rich plasma (PRP) gel has excellent tissue repair capabilities, especially vascular regenerative. It is a promising autologous derivative with applications in the treatment of diseases such as arthritis and tendon injuries 8, 9. PRP gel is produced by activating PRP, which is derived from whole blood, through the addition of calcium and thrombin 10, 11. Activated platelets rapidly degranulate, releasing high concentrations of growth factors (GFs), enriching the PRP gel with GFs such as platelet derived growth factor (PDGF) and insulin-like growth factors-1 (IGF-1) 12, 13. These properties enable PRP gel to regulate cellular processes such as migration, adhesion, proliferation, and differentiation while promoting extracellular matrix production and vascular regeneration, thereby facilitating tissue repair and reconstruction 14, 15. However, PRP gel application is limited by its low physical and thermal stability, rapid degradation, poor mechanical strength, and quick clearance of GFs 16, 17. Additionally, PRP contains low levels of neuroregenerative GFs such as nerve growth factor (NGF) and brain-derived neurotrophic factor (BDNF), which limits its application in the field of neural repair and regeneration. BDNF, a critical neurotrophic factor, promotes neural stem cell proliferation, induces their differentiation into neurons, and enhances axonal growth 18, 19. To address the limitation of PRP gel, we incorporated BDNF into the PRP gel to improve its neuroregenerative potential. This approach enables the PRP gel not only to support neural cell proliferation but also to induce their differentiation into neurons, thereby overcoming its limitations in neural repair and regeneration and achieving enhanced therapeutic efficacy.

Hydrogel is one of the ideal materials for cell growth and tissue regeneration after spinal cord injury. For example, Hao & Ni & Cui *et al.* prepared polyphenol-based chitosan hydrogels, which stabilize iron homeostasis after SCI and offer a promising strategy for SCI treatment 20. Considering the drawbacks of PRP, combining PRP gel with hydrogels addresses limitations such as poor stability and the rapid clearance of GFs 14. Selection of biosafe hydrogel materials with non-toxic synthesis processes and widely used in tissue engineering is more conducive to translation from the laboratory to the clinic. Sodium alginate (Alg), a naturally derived polysaccharide approved by the U.S. Food and Drug Administration (FDA), has the capacity to form hydrogels in the presence of divalent cations (e.g., Ca²⁺), creating an "egg-box" crosslinking model between adjacent guluronic acid blocks on the alginate chains 21. Therefore, Ca²⁺ can act as a cross-linking agent in the mixed PRP and Alg solution to establish a homogeneous interpenetrating network, and at the same time can activate the PRP hydrogel to release a large amount of growth factors to promote tissue repair and regeneration 21. This PRP and Alg double-network hydrogel with Ca²⁺ acting as both cross-linkers and activators shows great potential in wound healing 21. However, injectable PRP-Alg double-network hydrogels tend to be amorphous and have low modulus, making it difficult to provide structural support even though they can fill the SCI lesion site, which is not conducive to spinal cord regeneration. Additionally, Alg lacks cell adhesion sites, restricting its application in tissue engineering. Gelatin methacrylate (GelMA), derived from gelatin modified with methacrylate groups, forms photopolymerizable and patternable hydrogels while retaining gelatin's properties 22. It contains cell-extracellular matrix peptides which facilitates cell adhesion, such as the Arg-Gly-Asp (RGD) tripeptide sequence 23. Combining Alg with GelMA enhances both biocompatibility and mechanical properties, while also supplying cell adhesion sites for the hydrogel 24-26. Consequently, we designed a neural regeneration-enhanced triple-network hydrogel by combining GelMA with PRP, Alg, and BDNF. This hydrogel mimics the mechanical properties of spinal cord tissue, providing structural support and ensuring the sustained release of GFs and BDNF from PRP over time.

**Figure 1:**
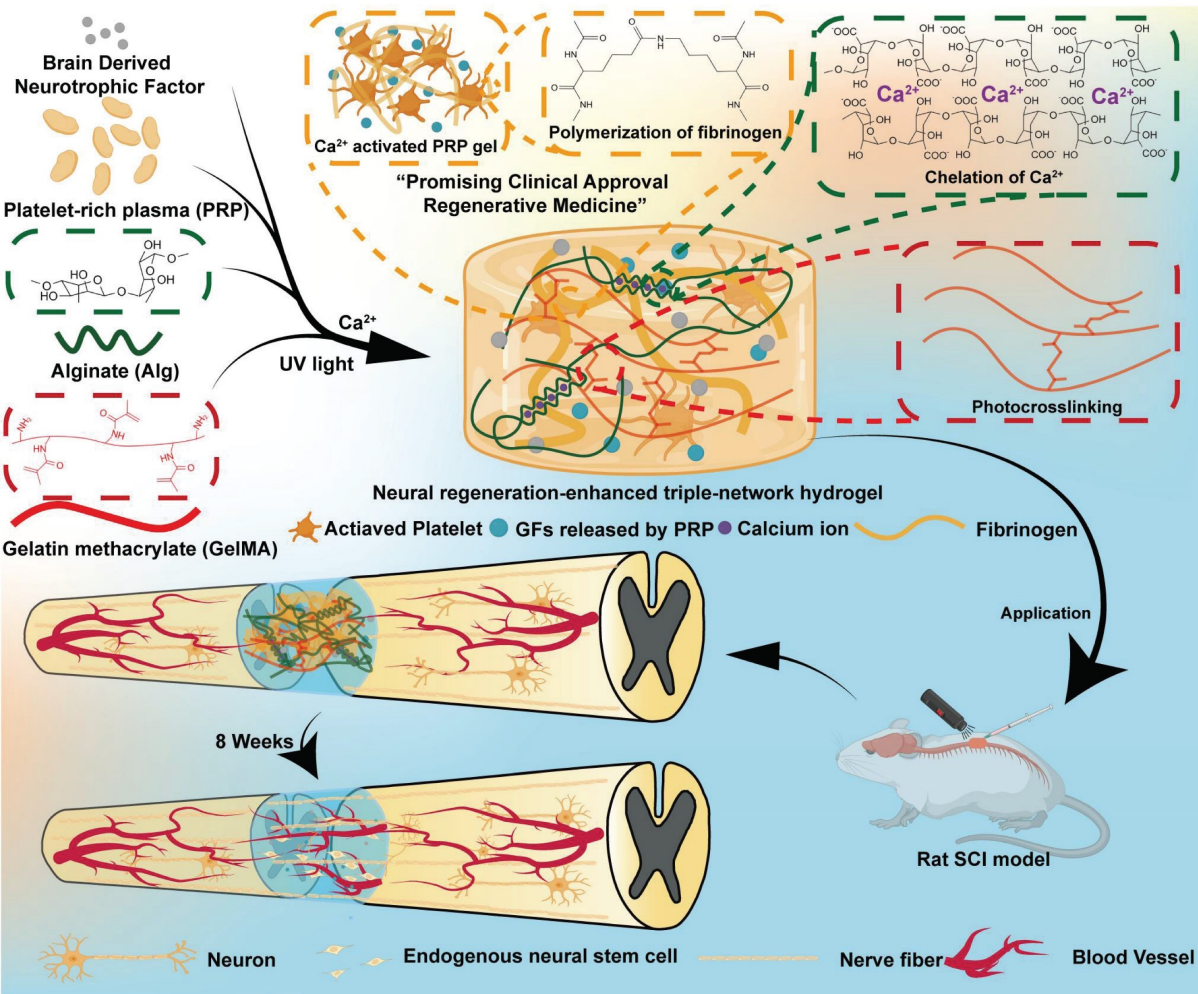
Schematic illustration of synthesis and application of the neural regeneration-enhanced triple-network hydrogels for SCI repair.

Therefore, we designed and developed a neural-enhancing triple-network injectable hydrogel to promote recovery in rats with complete spinal cord transection. Briefly, PRP, Alg, GelMA, and BDNF were pre-mixed. In the first step, Ca²⁺ were cleverly utilized as a cross-linking agent for the PRP-Alg interpenetrating double-network hydrogel. The third network, composed of GelMA, was then created via photopolymerization **(Figure 1)**. In this study, firstly, we cleverly used Ca²⁺ as a common cross-linking agent for the PRP gel network and the sodium alginate network, and the dual network hydrogel was injectable and able to adequately fill the defective area of the SCI before the third network was formed. Secondly, the formation of the third network confirmed that interpenetrating tri-network hydrogels with modulus, porosity, excellent biocompatibility, and slow-release function required for nerve cell growth were synthesized in this study. Third, the triple-network hydrogel is highly translatable due to its clever design and the FDA approval of the material in question. In conclusion, the PAG-BDNF hydrogel we developed has excellent dual biological functions of neural regeneration and angiogenesis, and it is a straightforward, viable, and promising therapeutic strategy for SCI regeneration.

## Results and Discussion

### Synthesis and physical characterization of neural regeneration-enhanced triple-network hydrogel

**Figure 2:**
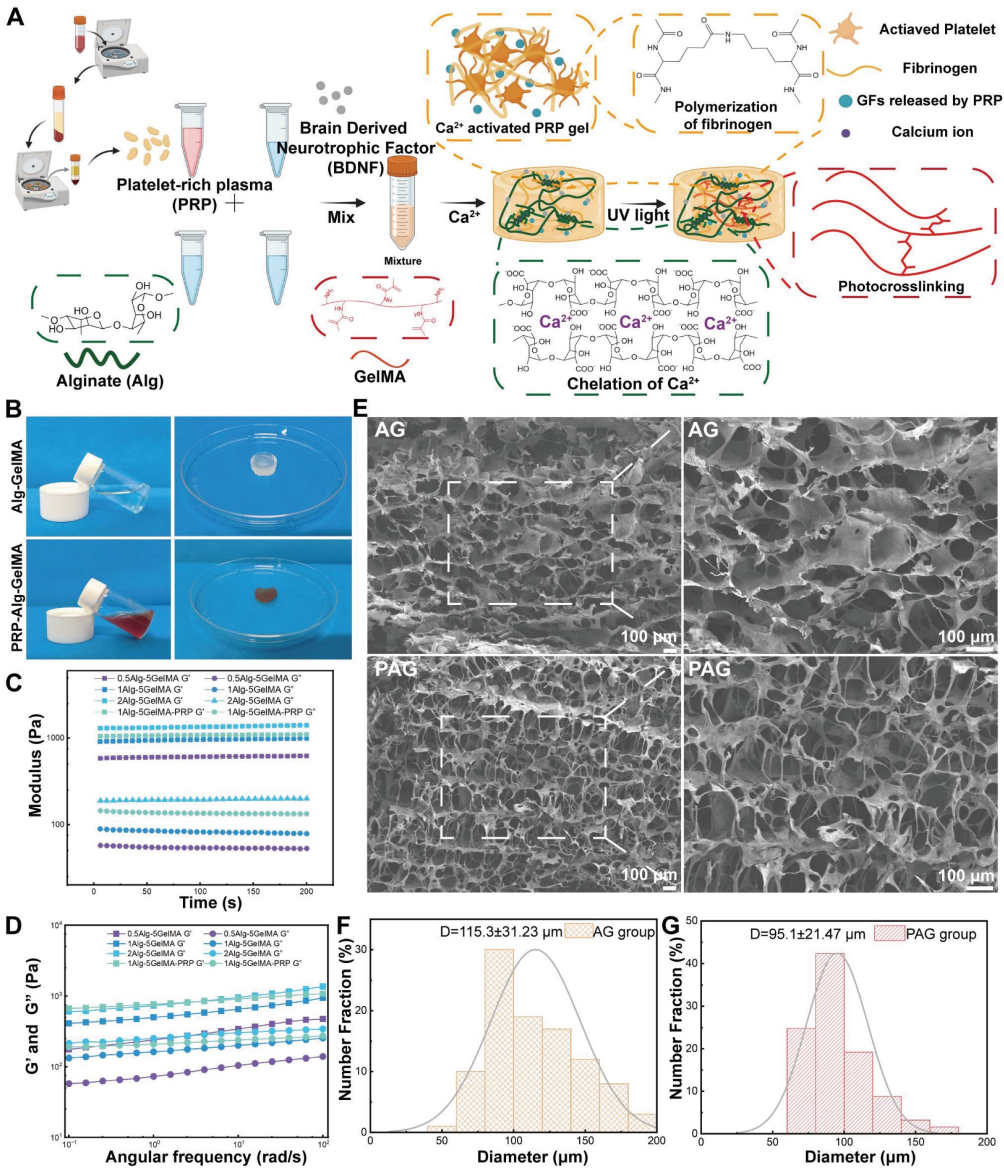
Characteristics of the neural regeneration-enhanced triple-network hydrogels. A) The synthesis process of the PAG-BDNF hydrogel (Figure was created with Biorender.com). B) Photographs show the mixing of Alg-GelMA (AG) and PRP-Alg-GelMA (PAG) hydrogels to form triple-network hydrogels. C). Time stability analysis of the hydrogels at a constant frequency of 1 Hz and 1% strain. D). Angular frequency sweep test (0.1-100 rad/s) conducted at a fixed strain of 1%. E). SEM images of AG and PAG hydrogels. F.G). The pore size distribution and average pore size of the AG and PAG hydrogels.

The use of bioactive hydrogels for *in situ* reconstruction of the damaged microenvironment has been proven to be an effective strategy for promoting SCI repair 27, 28. In this study, we developed a triple-network hydrogel using PRP, Alg, and GelMA. The synthesis process of the hydrogel is depicted in **Figure 2A**. Briefly, in the first step, Ca²⁺ serve both as an activator for the PRP gel and a crosslinker for Alg, resulting in the formation of a double-network hydrogel. In the second step, ultraviolet (UV) light is applied to crosslink the GelMA, forming the triple-network hydrogel **(Figure 2A)**. Whole blood was collected from Sprague-Dawley (SD) rats via cardiac puncture and processed through a two-step centrifugation to isolate PRP. The detailed methods described in the Methods section. **Figure 2B** shows the pre-gel mixtures of Alg-GelMA and PRP-Alg-GelMA, along with the macroscopic appearance of the triple-network hydrogels after the two-step gelation process.

The mechanical properties of hydrogels applied to SCI are a crucial factor in providing stable and effective support for neural growth *in vivo* 29. Studies have demonstrated that NSCs are more likely to differentiate into neurons when cultured in softer scaffolds (100-1000 Pa), while stiffer scaffolds (7-10 kPa) tend to promote astrocytic differentiation 30, 31. In the triple-network hydrogels developed in this study, the mechanical characteristics are mainly governed by the covalent and non-covalent interactions within the system. The covalent network is formed by GelMA through free radical polymerization, resulting in a covalent crosslinked structure, while the non-covalent interactions involve the formation of triple helical networks through hydrogen bonding among GelMA chains and the crosslinking of sodium alginate by Ca²⁺ 32. Notably, Ca²⁺ not only crosslink the sodium alginate but also activate the PRP network. In a previous study, NSCs were more suitable for growth and differentiation in 5% GelMA compared to 10% GelMA 29. Therefore, we first evaluated the rheological properties of hydrogels with 0.5%, 1%, and 2% Alg and 5% GelMA. At 25 °C, we measured the time stability of the hydrogels at a fixed frequency of 1 Hz and 1% strain **(Figure 2C)**. The storage moduli (G') of 0.5% Alg-5% GelMA, 1% Alg-5% GelMA, and 2% Alg-5% GelMA were 589.7 ± 10.6 Pa, 929.9 ± 22.9 Pa, and 1311.3 ± 32.8 Pa, respectively. Compared to the 0.5% Alg-5% GelMA and 2% Alg-5% GelMA hydrogels, the G' of the 1% Alg-5% GelMA hydrogel was approximately 1000 Pa, similar to the range of natural spinal cord tissue 33-36. Subsequently, under a constant oscillatory strain of 1%, as the angular frequency increased from 0.1 rad/s to 100 rad/s, the G' of the hydrogel consistently exceeded the loss modulus (G'') **(Figure 2D)**. This outcome indicates that the hydrogel demonstrates good stability across this range of oscillatory frequencies. These rheological results suggest that the modulus of these hydrogels is in the range favorable to support the differentiation of NSCs towards neurons. Hydrogels with a modulus of around 1 kPa have been shown to significantly enhance tissue regeneration in previous studies 37, 38. In addition, we hope that the hydrogel will also provide effective structural support in the area of the SCI defect. Taking these factors into account, we selected the 1% Alg-5% GelMA (AG) hydrogel for further experiments.

**Figure 3:**
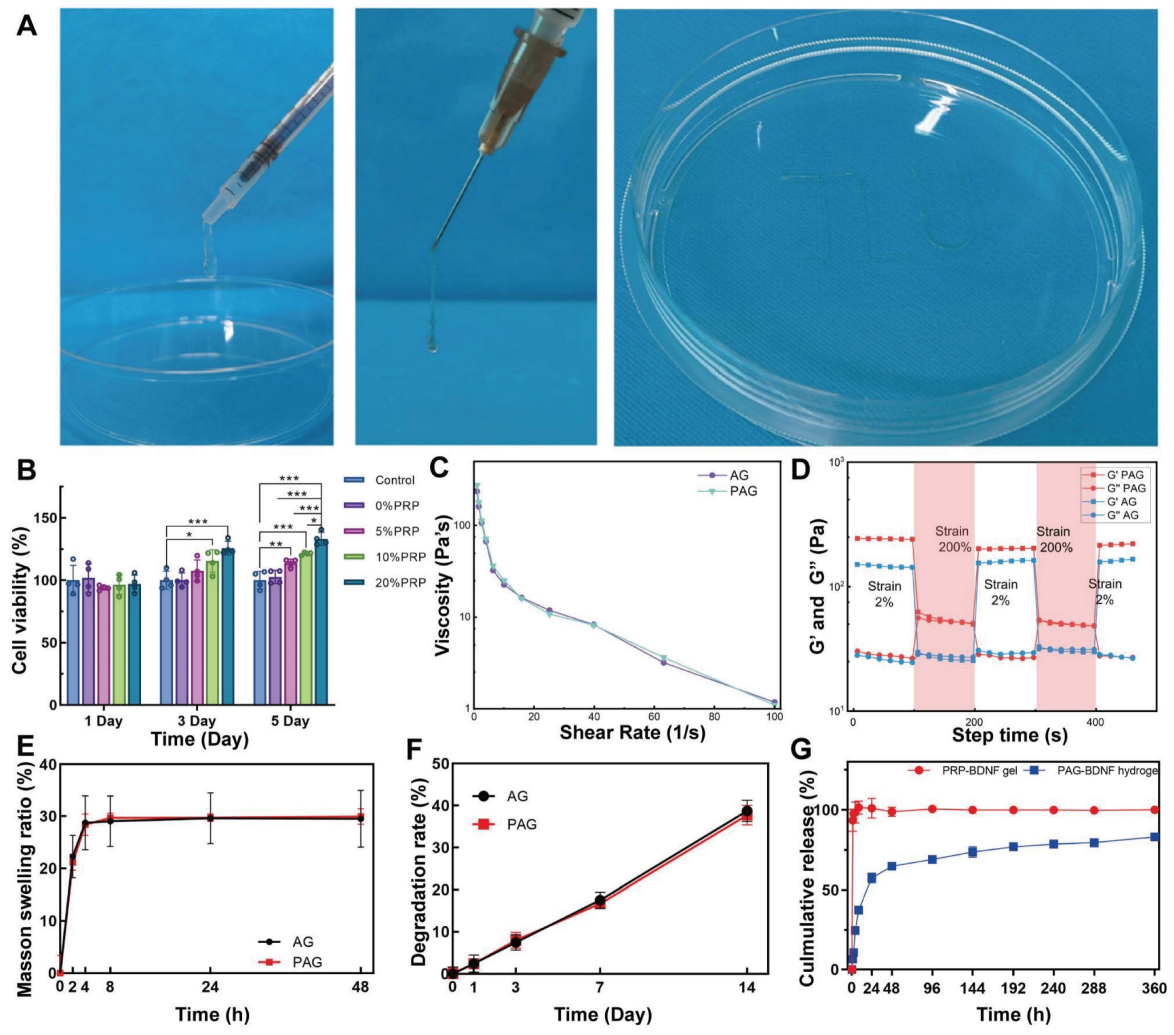
A). The injection of AG and PAG hydrogel through a syringe. B). CCK-8 results of HUVECs in hydrogels containing 5%, 10%, and 20% PRP at day 1, day 3 and day 5 (n = 4 per group). C). Viscosity measurement of AG and PAG hydrogels. D). Storage (G′) and loss (G″) moduli of AG and PAG hydrogels after Ca^2+^ crosslinked under varying recycled strain. E). Swelling ratios and F) degradation profiles of different hydrogels in phosphate-buffered saline (n = 3 per group). G). The release profiles of protein from the PRP-BDNF gel and PAG-BDNF (n = 3 per group). Error bars indicate the SD (*P < 0.05, **P < 0.01, ***P < 0.001).

Next, we investigated the optimal PRP concentration to synthesize a triple-network hydrogel best suited for tissue regeneration. In our pre-experiment, after UV irradiation of 30 s 30% PRP-Alg-GelMA hydrogel, although its exterior forms hydrogel but its interior is still in sol-gel state. In contrast, hydrogels with PRP concentrations of 5%, 10%, and 20% formed hydrogels well inside and outside after 30 s of UV irradiation. In order to ensure the consistency of the hydrogel synthesis process, we cultured HUVECs in hydrogels containing 5%, 10%, and 20% PRP to evaluate their effects on promoting cell proliferation. The results of CCK-8 assay indicated that the 20% PRP hydrogel showed the best proliferation-promoting effect compared to the other groups **(Figure 3B)**. Additionally, the G' of the 20% PRP-1% Alg-5% GelMA hydrogel (1079.28 ± 13.1 Pa) and the 1% Alg-5% GelMA hydrogel (929.9 ± 22.9 Pa) were closest to 1 kPa, which is beneficial for nerve cell proliferation. Therefore, we selected the 20% PRP-1% Alg-5% GelMA (PAG) hydrogel for subsequent experiments.

After determining the optimal PRP concentration, we further examined the morphological characteristics of AG and PAG hydrogels under an electron microscope. **Figure 2E** showed that both AG and PAG hydrogels exhibited irregular shapes and a loose, porous structure with variable pore sizes after freeze-drying. The average pore size of the AG and PAG hydrogels were 115.3 ± 31.23 and 95.1 ± 21.47 μm respectively **(Figure 2F,2G)**. This structure enhances cell adhesion and facilitates cell infiltration 39, 40. In comparison, PAG hydrogels showed more filamentous cross-links than AG, likely due to the presence of PRP gel. Overall, the triple-network hydrogels PAG displayed an interconnected and evenly distributed structure at the microscale, which is favorable for drug release and cell migration 37.

Considering the mobility of the spine and the defective environment of SCI, hydrogels with injectable as well as self-healing properties are more suitable for the treatment of spinal cord injuries. For example, Ni & Sun *et al.* designed an injectable multifunctional hydrogel to fill and support the irregular injury region of SCI through covalent and non-covalent interactions between carboxymethyl chitosan and gallic acid, providing a paradigm for injectable hydrogels applied to the complex environment of SCI 41. In this paper, the injectability of the hydrogel was assessed using a 1 mL syringe equipped with a 0.5 mm diameter needle. Since our strategy is to inject the hydrogel into the SCI region before UV crosslinking, we test the injectability of the hydrogel after Ca^2+^ crosslinking. After the first step of Ca^2+^ cross-linking, both AG and PAG hydrogels were able to be injected through the needle, even forming the letters "JLU" demonstrating their injectability **(Figure 3A)**. Additionally, we observed a change in the hydrogel's viscosity in response to varying shear rates. As illustrated in **Figure 3C**, the viscosity of the hydrogel decreased significantly as the shear rate increased. This confirmed that the hydrogels exhibit shear-thinning abilities, allowing them to be injected into areas with complex geometric defects, thereby effectively filling SCI sites. The application scenario of the hydrogel in this study was first Ca^2+^ crosslinking to form a PRP-Alg hydrogel, which was subsequently injected into the SCI injury area, and finally UV irradiation was performed. Considering the above application scenarios, we conducted oscillatory shear tests on Ca^2+^ crosslinked injected hydrogels. During this continuous testing process, the hydrogel transitioned from a gel to a sol state (G' < G'') under high oscillatory strain (200%). When the strain was reduced back to a low level (2%), the hydrogel reverted from a sol to a gel state (G' > G''), demonstrating its self-healing capability **(Figure 3D)**. This indicates that the hydrogel can maintain stability after injection into the injury site, providing effective support to the damaged area 42, 43. Previous studies have shown that GelMA hydrogels, by themselves, lack self-healing properties. In this study, Alg provides sacrificial bonds to the composite hydrogels, conferring self-healing properties to the hydrogels after UV crosslinked** (Figure S1)**. Therefore, the incorporation of supramolecular Alg provides self-repair capabilities to the triple-network hydrogel 44.

The swelling and degradation properties of hydrogels are critical to their effectiveness in tissue engineering applications 45, 46. Our results revealed that the swelling rates of AG and PAG hydrogels reached 28.7 ± 3.6% and 28.4 ± 0.8%, respectively, after 4 hours, with maximum swelling ratios of approximately 29.5 ± 3.3% and 29.9 ± 1.1% after 48 hours **(Figure 3E)**. This relatively low swelling ratio indicates reduced deformation, which contributes to providing stable support for regeneration at the SCI injury site. The degradation results for each hydrogel group demonstrated that as the hydrogels gradually degraded, they provided a stable microenvironment and long-term support for the injured area 47. In PBS, the AG and PAG hydrogels degraded by 38.7 ± 2.5% and 37.7 ± 2.3% after 14 days** (Figure 3F)**. This suggests that the hydrogels can provide long-term and stable support to the SCI injury site. Subsequently, the protein release profiles of PRP-BDNF gel and PRP-Alg-GelMA-BDNF (PAG-BDNF) hydrogel were then evaluated using the BCA protein assay kit. As depicted in **Figure 3G**, the PRP-BDNF gel exhibited an initial burst release of 93.4 ± 6.5% within the first hour, while PAG-BDNF triple-network hydrogels released 64.9 ± 1.2% over a span of 48 hours. The release rate then gradually slowed, extending to 15 days with a release of 83 ± 1.3%. This suggests that the triple-network hydrogels are capable of continuous and sustained GFs release, achieving controlled release beneficial for the long-term treatment of SCI. During the synthesis of the hydrogels in this study, firstly PRP was homogeneously mixed with Alg and GelMA, and subsequently the addition of Ca^2+^ activated the formation of a dual network PRP-Alg hydrogel. At the same time, Ca^2+^ activated PRP to release the internal bioactive substances uniformly dispersed in the PRP-Alg dual-network hydrogel. The bioactive substances released by PRP were completely encapsulated by the PRP-Alg hydrogel, and the subsequent cross-linking of the GelMA network became the third network of the composite hydrogel. All of the biologically active substances released from the encapsulated PRP were released with the degradation of the hydrogel. Although Ca^2+^ crosslinked Alg hydrogels when in PBS, divalent cations gradually replace monovalent cations (Na^+^) in the surrounding medium due to exchange reactions and stimulate alginate dissolution. However, in this study, the presence of the GelMA third network allowed the structure of the composite hydrogel to be maintained for a long period of time and it did not disintegrate even after 14 days of immersion in PBS **(Figure 3F)**. Previous studies have shown that when Alg-GelMA hydrogel was immersed in PBS, the hydrogel remained undegraded up to 28 days, although the phosphate in PBS binds the Ca^2+^ to the alginate and leads to partial degradation of the hydrogel 48. In another experiment, GelMA/Alg hydrogels immersed in PBS lasted until 21 days without disintegration 49. Therefore, we speculate that in our experiments, the third network, GelMA network, was able to maintain the integrity of the composite hydrogel without rapid disintegration, even with Alg dissolution due to displacement of Ca^2+^. In conclusion, the PAG-BDNF triple-network hydrogel not only mitigates the burst release issue associated with PRP gel but also provides extended GF delivery to the SCI region over time.

Overall, in this part we successfully synthesized and characterized the PAG-BDNF triple-network hydrogel, while several characterization results showed that this hydrogel has properties such as modulus suitable for neural cell growth, porous structure conducive to cell adhesion, migration and drug release, injectability, self-repairing, degradability, and long-term sustained release. These indicate that it is capable of sustained release of GFs while supporting the injured area of SCI over a long period of time, achieving a controlled release that is beneficial to the long-term treatment of SCI.

### Biocompatibility of the neural regeneration-enhanced triple-network hydrogel and its effects on the proliferation and adhesion of HUVECs

**Figure 4:**
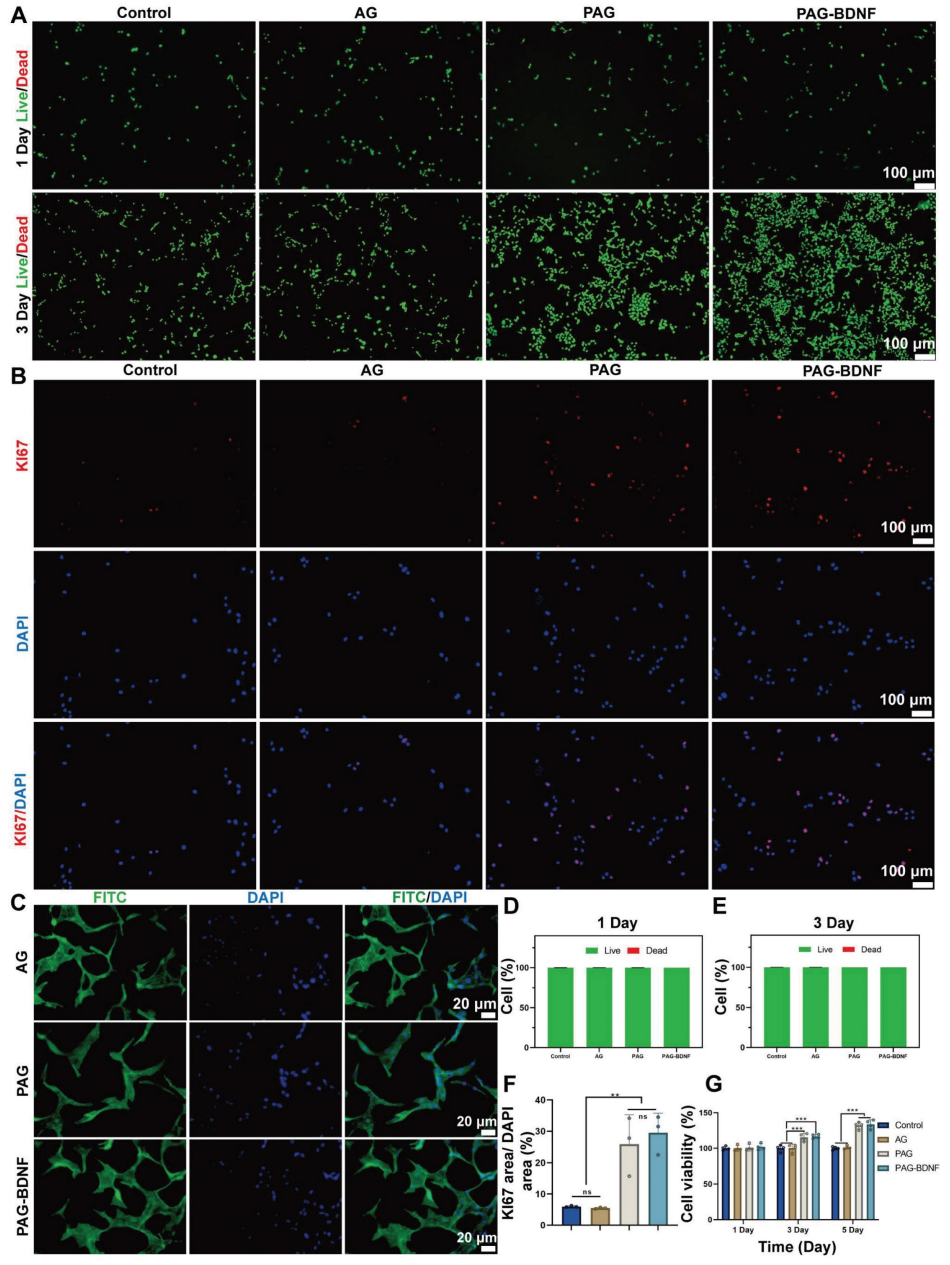
Biocompatibility of hydrogels *in vitro* and effects on proliferation and adhesion of HUVECs. A). Live/dead staining results of HUVECs from Control (PBS), AG, PAG, PAG-BDNF and their D-E) quantitative data (n = 3 per group). B). Representative Ki67 immunofluorescence images of different groups and their F) quantitative data (n = 3 per group). C). FITC-phalloidin (Green) and DAPI (Blue) staining results of AG, PAG, PAG-BDNF. G). CCK-8 results of HUVECs at day 1, day 3 and day 5 (n = 4 per group). Error bars indicate the SD (*P < 0.05, **P < 0.01, ***P < 0.001).

Excellent biocompatibility is fundamental to tissue engineering materials 50. Live/Dead staining was used to assess the effect of Control (PBS), AG, PAG and PAG-BDNF hydrogels on the biocompatibility of HUVECs. No significant cell death was observed after culturing HUVECs on different hydrogels for 1 and 3 days** (Figure 4A)**. Quantitative data in **Figure 4D-E** show that the survival rates in the Control, AG, PAG, and PAG-BDNF groups exceeded 95% at both time points, indicating very low cytotoxicity and confirming the excellent biocompatibility of the hydrogels. The effects of the hydrogels on HUVEC proliferation were further evaluated using the CCK-8 assay and Ki67 staining. As illustrated in **Figure 3G**, the absence of bioactive substances in the AG group (99.9 ± 4.1%, 100.2 ± 45.9%, and 101.2 ± 2.6%) resulted in no discernible impact on cell proliferation at days 1, 3, and 5, comparable to the control group (99.9 ± 2.9%, 100 ± 4.8%, and 100 ± 2.5%) (P > 0.05). Previous studies have shown that PRP releases a large amount of GFs upon activation, which strongly promotes cell proliferation 51. Our results confirmed this, with the PAG and PAG-BDNF groups exhibiting significantly elevated proliferation on days 3 (115.4 ± 5.4% and 116.8 ± 2.8%) and 5 (132.2 ± 4.9% and 133.8 ± 6.2%) in comparison to the Control and AG groups (*P* < 0.05). However, no statistically significant discrepancy was discerned between the PAG and PAG-BDNF groups. Ki67 is a protein strongly associated with cell proliferation, and its expression level correlates with mitotic activity 52. As shown in **Figure 4B**, after 24 hours, the PAG and PAG-BDNF groups exhibited higher Ki67 expression compared to the Control and AG groups. The quantitative results in **Figure 4F** further confirmed this, with the PAG (25.9 ± 9.4%) and PAG-BDNF (29.5 ± 6.3%) groups showing higher proliferation compared to the Control (6 ± 0.2%) and AG (5.5 ± 0.3%) groups, demonstrating the triple-network hydrogel's strong ability to promote HUVEC proliferation (*P* < 0.01). Cell adhesion is another critical requirement for tissue engineering hydrogels. We used FITC-phalloidin to observe the effects of hydrogels on HUVEC adhesion. The **Figure 4C** showed that HUVECs were able to spread and extend filopodia on all hydrogel groups, indicating that the hydrogels promote cell adhesion. This effect is attributed to the presence of GelMA, which provides adhesion sites, enhancing the adhesive properties of the hydrogels.

The results of this part of the experiment demonstrated that the composite hydrogel exhibited excellent biocompatibility, adhesion properties, and HUVECs proliferation-promoting ability when co-cultured with HUVECs cells, and that this strong HUVECs proliferation-promoting effect was mainly attributed to the incorporation on PRP.

### The effects of the neural regeneration-enhanced triple-network hydrogel on angiogenesis

**Figure 5:**
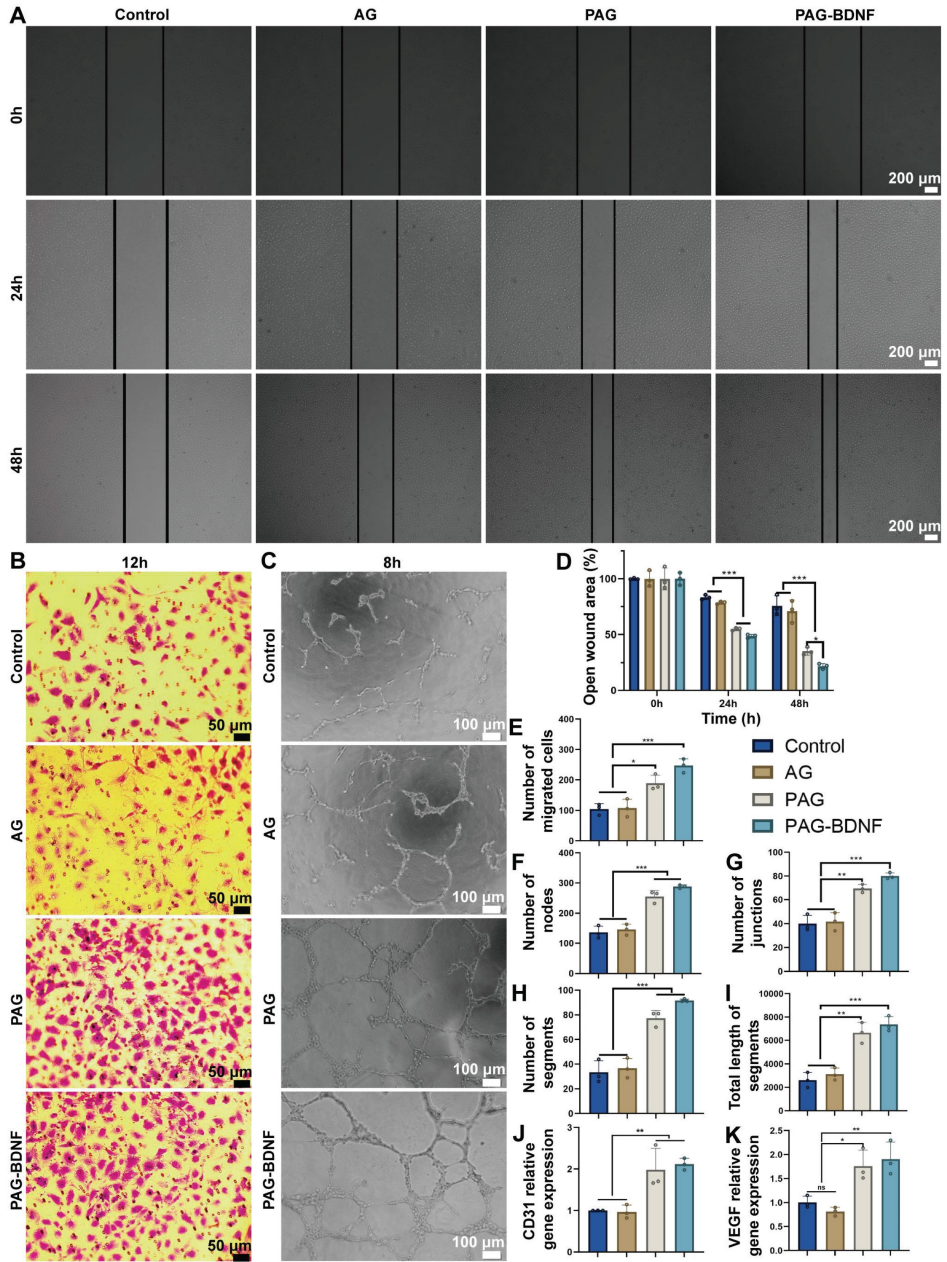
The effects of hydrogels on wound healing assay, cell invasion assay and tube formation assay on HUVECs. A). Wound healing assay results of Control (PBS), AG, PAG and PAG-BDNF with HUVECs and D) their quantitative analysis (n = 3 per group). B). Cell invasion assay results of Control, AG, PAG and PAG-BDNF with HUVECs and E) their quantitative analysis (n = 3 per group). C). Tube formation assay results of Control, AG, PAG and PAG-BDNF with HUVECs and their quantitative analysis on F) the number of segments, G) number of nodes, H) number of junctions, and I) total length of segments (n = 3 per group). J) and K) qPCR analysis of VEGF and CD31 genes (n = 3 per group). Error bars indicate the SD (*P < 0.05, **P < 0.01, ***P < 0.001).

Prior research has demonstrated that PRP gel, comprising GFs, can facilitate enhanced cell proliferation, cell migration, and angiogenesis 16, 53-55. The wound healing assay was employed to assess the effects of different hydrogels on the migration capacity of HUVECs. The light microscopy images **(Figure 5A)** and quantitative results **(Figure 5D)** demonstrated that, following a 48-hour period, the AG group (71 ± 9.95%) did not exhibit enhanced cell migration in comparison to the control group (75.8 ± 9.01%) (P > 0.05). In contrast, the PAG group (35.3 ± 3.5%) and the PAG-BDNF group (21.8 ± 2.42%) exhibited significantly stronger cell migration. The capacity of the cells to migrate was significantly greater in the PAG-BDNF group than in the PAG, AG and Control groups. These findings indicate that PRP and BDNF can facilitate the migration of HUVECs.

The cell invasion assay was employed for the further evaluation of the impact of the hydrogels on the migration capacity of the HUVECs. As illustrated in** Figure 5B**, the incorporation of PRP and BDNF into hydrogels resulted in a notable increase in the number of cells that traversed the polycarbonate membrane, as observed in both PAG and PAG-BDNF. The number of migrated cells in the PAG-BDNF (246.67 ± 23.03) and PAG (189.33 ± 25.81) groups were found to be significantly higher than in the Control (104.33 ± 18.18) and AG groups (107.68 ± 29.54) (P < 0.05). Although the number of cells migrating in the PAG-BDNF group was more than that in the PAG group, there was no statistical difference between the two groups. This suggests that the incorporation of PRP and BDNF has augmented the chemotactic characteristics of the hydrogel.

The capacity of each sample group to facilitate HUVEC tubule formation was evaluated through the utilization of a tube formation assay. As illustrated in **Figure 5C**, the formation of intact tubules was less prevalent in the Control and AG groups. However, the PAG and PAG-BDNF groups exhibited the formation of dense and intact tubules. Quantitative analysis was conducted on the number of nodes, number of segments, number of junctions, and total length of segments. **Figures 5F-I** show that there was no statistically significant difference between the Control and AG group (P > 0.05). The PAG-BDNF and PAG groups demonstrated a markedly higher number of nodes, segments, junctions, and total segment lengths in comparison to the Control and AG groups. There was no statistical difference between the PAG-BDNF and PAG groups. These findings indicate that the AG hydrogel devoid of PRP and BDNF possesses a constrained capacity to stimulate tubule formation in HUVECs. PRP and BDNF demonstrate strong capabilities in promoting tubule formation in HUVECs.

To further evaluate the effect of each hydrogel on angiogenesis, quantitative polymerase chain reaction (qPCR) assays was conducted on HUVECs co-cultured with each hydrogel to assess the expression levels of the angiogenic genes platelet endothelial cell adhesion molecule (CD31) and vascular endothelial growth factor (VEGF) 56. As shown in **Figures 5J-K**, the expression levels of CD31 and VEGF in the PAG-BDNF and PAG group were markedly elevated in comparison to AG and Control groups. In summary, these results demonstrate that the PAG and PAG-BDNF hydrogels significantly enhanced vascularization *in vitro*, which is attributed to the ability of one of them, PRP, to strongly promote vascular regeneration itself.

### The biocompatibility of the neural regeneration-enhanced triple-network hydrogel with NSCs and its effect on their proliferation

**Figure 6:**
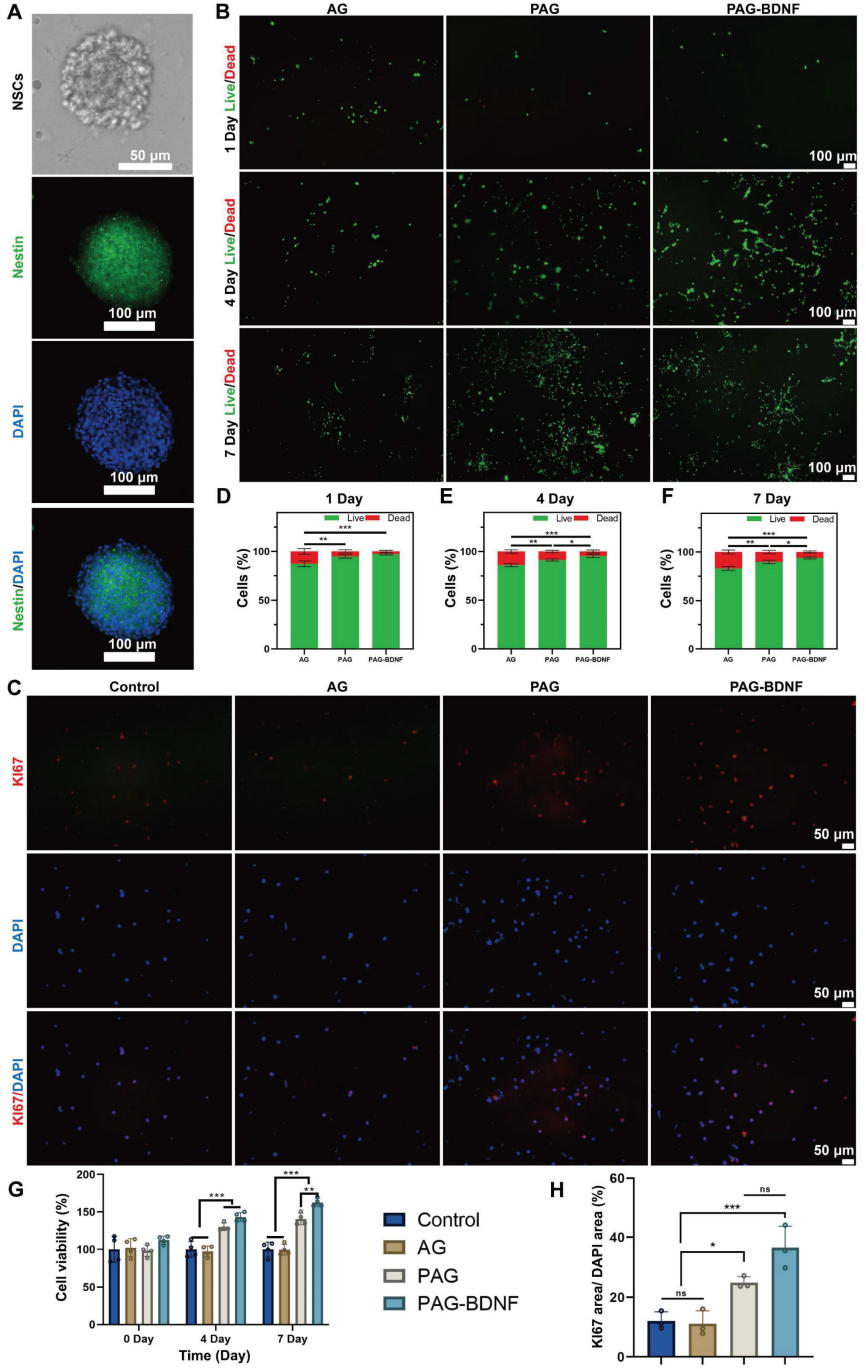
Biocompatibility of hydrogels *in vitro* and effects on proliferation of NSCs. A). The characteristics of the extracted NSCs under light microscopy and confocal microscopy. B). Live/dead staining results of NSCs from different groups and their D-F) quantitative data (n = 3 per group). C). Representative Ki67 immunofluorescence images of different groups and their H) quantitative data (n = 3 per group). G). CCK-8 results of NSCs at day 1, day 4 and day 7 (n = 4 per group). Error bars indicate the SD (*P < 0.05, **P < 0.01, ***P < 0.001).

Nestin is an intermediate filament protein that is specifically expressed by NSCs, and immunofluorescence staining was employed for the purpose of identifying fetal mouse NSCs 57. **Figure 6A** shows the characteristics of the extracted NSCs under light microscopy and immunofluorescence. The results demonstrated that all extracted cells exhibited positive Nestin expression, thereby confirming that the isolated cells were NSCs. Next, we assessed the biocompatibility of the hydrogel with NSCs using Live/Dead staining. Fluorescence microscopy results showed that NSCs cultured on different hydrogel groups for 1, 4, and 7 days did not exhibit a large number of dead cells. Quantitative results revealed that AG, PAG, and PAG-BDNF hydrogels had a survival rate of over 90%, indicating excellent biocompatibility **(Figures 6B, 6D-F)**. We then evaluated the effects of the hydrogel on NSCs proliferation using the CCK-8 and Ki67 staining. As illustrated in **Figure 6G**, the CCK-8 results demonstrated that the AG hydrogel, which lacks bioactive substances, had no notable impact on NSCs proliferation in comparison to the control group. However, due to the addition of PRP, both PAG and PAG-BDNF groups exhibited strong proliferative effects. From day four onward, the survival and proliferation of NSCs in the PAG and PAG-BDNF groups remained consistently higher. Thanks to the addition of BDNF, the PAG-BDNF group showed superior performance compared to the PAG group, exhibiting the most significant effect on NSCs proliferation. Ki67 staining results were consistent with the CCK-8 results, with PAG-BDNF having the highest Ki67 expression **(Figure 6C)**. Quantitative analysis in **Figure 6H** showed that PAG-BDNF (36.5 ± 7.2%) outperformed PAG (25 ± 2%), AG (11.1 ± 4.3%), and the Control (12.1 ± 3%). These results indicate that the hydrogel system possesses excellent biocompatibility. Furthermore, due to the addition of PRP and BDNF, the PAG-BDNF hydrogel showed the greatest effect in promoting NSCs proliferation.

The results of this part of the experiment showed that the composite hydrogel showed excellent biocompatibility and NSCs proliferation-promoting ability after co-culture with NSCs, and the addition of BDNF enhanced the effect of PRP in promoting the proliferation of NSCs. This result is different from the above results after co-culture with HUVECs, which suggests that BDNF plays a great role in the proliferation of NSCs, while its effect is not obvious in HUVECs.

### The effects of the neural regeneration-enhanced triple-network hydrogels on NSCs differentiation and migration

**Figure 7:**
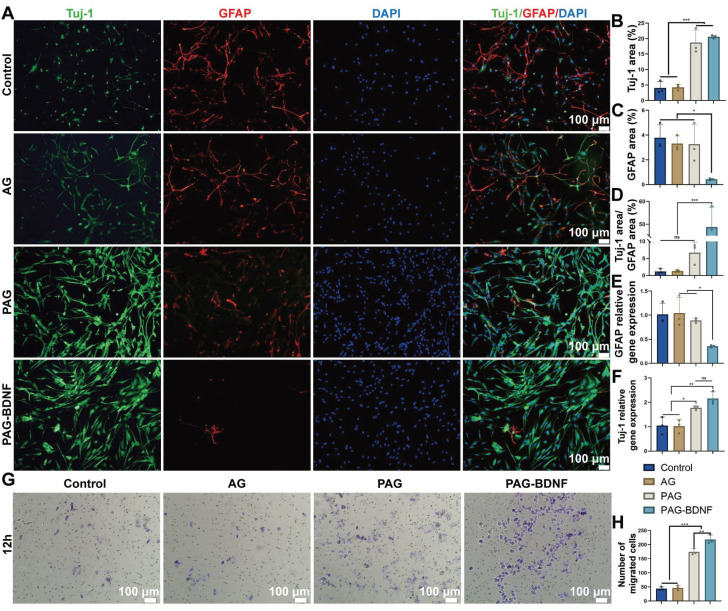
*In vitro* NSCs differentiation and cell invasion assay on the neural regeneration-enhanced triple-network hydrogels. A). Immunofluorescence images of NSC differentiation on different groups for 7 days. Neurons (green), astrocytes (red), DAPI (blue). B), C) and D). The illustration depicts the quantitative analysis of Tuj-1, GFAP, Tuj-1/GFAP areas (n = 3 per group). D and E). qPCR analysis of Tuj-1 and GFAP genes (n = 3 per group). G). Cell invasion assay results of Control (PBS), AG, PAG and PAG-BDNF with NSCs and H) their quantitative analysis (n = 3 per group). Error bars indicate the SD (*P < 0.05, **P < 0.01, ***P < 0.001).

NSCs are capable of differentiating into neurons and astrocytes, which are identified by beta-tubulin III (Tuj-1) and glial fibrillary acidic protein (GFAP), respectively 58. In the treatment of SCI, promoting the differentiation of NSCs into neurons is crucial. Next, we examined the effects of different groups on NSCs differentiation 59, 60. **Figure 7A** indicates that the PAG and PAG-BDNF groups exhibited a significantly higher number of Tuj-1 positive neurons compared to the Control and AG groups (*P* < 0.05). In contrast, the expression of GFAP was markedly diminished in the PAG-BDNF group in comparison to the Control, AG, and PAG groups (P < 0.05). Quantitative results shown in **Figure 7B-D** align with the fluorescence images. A significant difference was observed in the size of the Tuj-1 positive area between the PAG-BDNF (20.59 ± 0.49%) and PAG (18.74 ± 4.06%) groups in comparison to the Control (4.08 ± 2.04%) and AG (4.27 ± 0.87%) groups. Meanwhile, the GFAP positive area was significantly lower in the PAG-BDNF group (0.43 ± 0.07%) compared to the Control (3.79 ± 1.04%), AG (3.31 ± 0.61%), and PAG (3.26 ± 1.57%) groups. Notably, **Figure 7D** shows that the Tuj-1/GFAP ratio in the PAG-BDNF group was significantly higher than in the other three groups (*P* < 0.001). However, there was no statistical difference among the PAG, Control, and AG groups, suggesting that PRP alone did not significantly affect neuronal differentiation. The incorporation of BDNF augmented the capacity of PRP to facilitate neuronal directional differentiation. To further investigate NSCs differentiation, qPCR analysis was performed to assess the expression of neuron-related and astrocyte-related genes in different groups of NSCs. The results were consistent with the fluorescence findings. After 7 days of culture, Tuj-1 expression levels in the PAG-BDNF and PAG groups were higher than in the AG and Control groups, while GFAP expression was significantly lower in the PAG-BDNF group compared to the Control, AG, and PAG groups **(Figure 6E-F)**. In summary, these results indicate that PRP can promote NSCs differentiation, although this differentiation lacks directionality. The addition of BDNF effectively restricts the differentiation of NSCs into astrocytes, providing strong support for neuronal regeneration following SCI. In addition, the addition of BDNF improved the disadvantage of PRP in neural tissue repair and informed the clinical application of PRP in nerve repair in the future.

Materials are particularly important for the ability to recruit endogenous NSCs after SCI. The cell invasion assay was employed for the further evaluation of the impact of the hydrogels on the migration capacity of the NSCs. As illustrated in Figure 6G, the incorporation of PRP and BDNF into hydrogels resulted in a notable increase in the number of cells that traversed the polycarbonate membrane, as observed in both PAG and PAG-BDNF. The number of migrated cells in the PAG-BDNF (218 ± 15.72) groups were found to be significantly higher than in the Control (44 ± 6.08), AG (46.67 ± 8.5) and PAG groups (174.33 ± 11.15) (*P* < 0.05) (**Figure 7H**). This suggests that the incorporation of BDNF has augmented the chemotactic characteristics of the PRP to NSCs This implies that the hydrogel is beneficial in promoting the recruitment of endogenous NSCs after implantation in SCI rats.

The results in this part show that although PAG hydrogel promotes the differentiation of NSCs to neurons, it equally promotes the differentiation of NSCs to astrocytes, which is detrimental to the repair of spinal cord injury 61. With the addition of BDNF, the PAG-BDNF hydrogel altered these results. It strongly promoted the differentiation of NSCs to neurons and reduced the expression of GFAP. This reduced the possibility of astrocytes forming a glial scar barrier to hinder nerve regeneration.

### Neural regeneration-enhanced triple-network hydrogel implantation promotes motor function recovery and improves neural pathology in SCI rats

**Figure 8:**
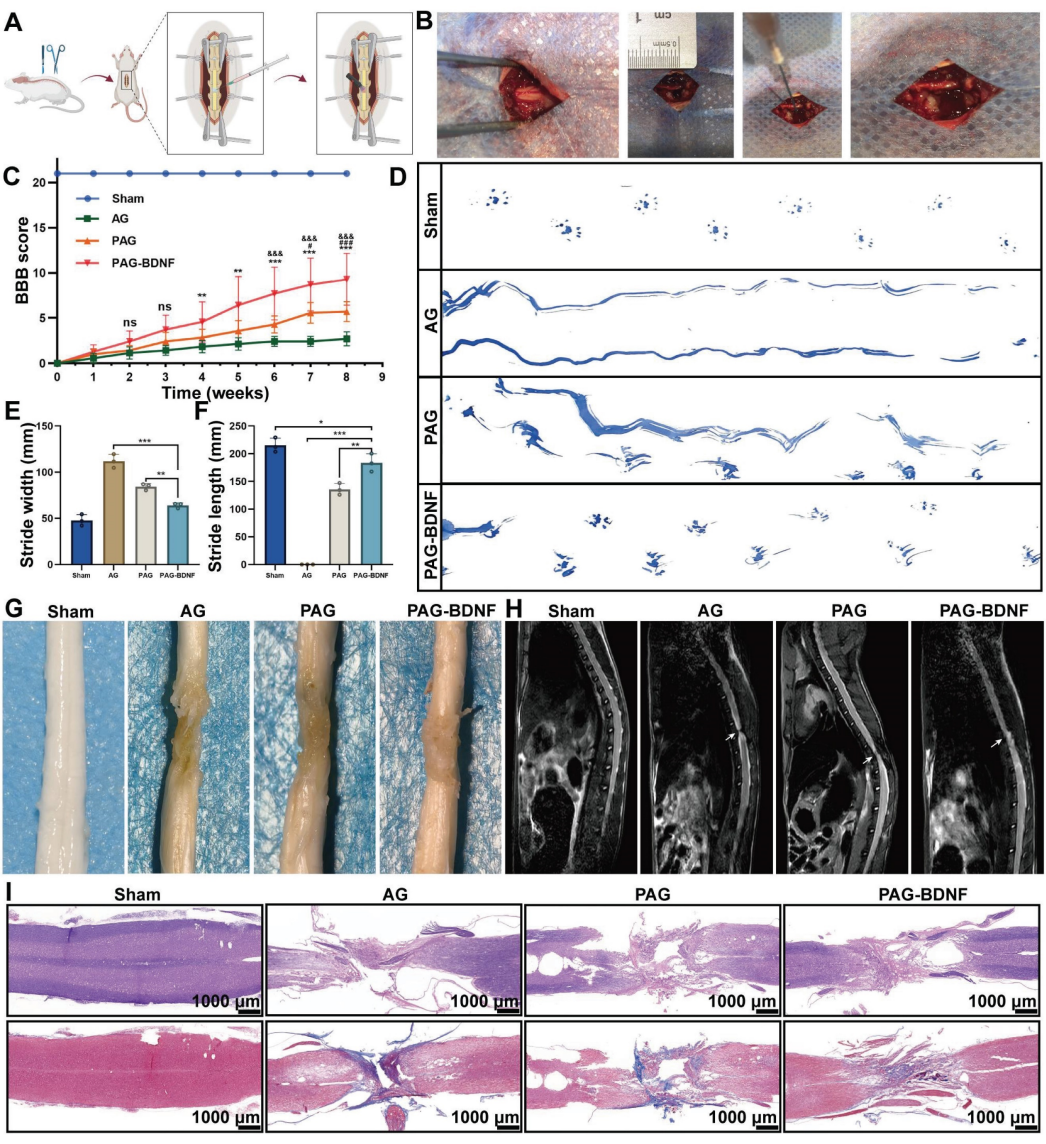
PAG-BDNF hydrogel improves motor function recovery and improves neural pathology. A-B). The illustration depicts the process involved in the traumatic SCI and hydrogel injection procedure. C). The recovery of motor function in rats was evaluated using the BBB score on an open field. (AG vs. PAG-BDNF, **P < 0.01, ***P < 0.001; AG vs. PAG, #*P* < 0.05, ##*P* < 0.01, ###*P* < 0.001; PAG vs. PAG-BDNF, &*P* < 0.05, &&*P* < 0.01, &&&*P* < 0.001, Sham group n = 6, AG, PAG, PAG-BDNF groups n = 7). D). A representative footprint analysis was employed to evaluate the recovery of hind limb motor function in rats from the various experimental groups. The hind paw was stained with blue ink. E). Stride width and F) length were used to quantify locomotion recovery at 8 weeks after injury (n = 3 per group). (*P < 0.05, **P < 0.01, ***P < 0.001). G). Representative spinal cord images of Sham, AG, PAG and PAG-BDNF at 8 weeks. H). Representative MRI images after SCI repair in Sham, AG, PAG and PAG-BDNF. White arrows indicate the lesion area of the SCI. J). Representative images of H&E and Masson staining show the morphology of the spinal cord in Sham, AG, PAG, PAG-BDNF.

In order to evaluate the therapeutic effects of AG, PAG and PAG-BDNF on SCI, a 2mm spinal cord transection model was established in SD rats. After cross-linking the hydrogel with Ca^2+^, we injected it into the lesion site, and a 20s UV exposure was used to form the triple-network hydrogel **(Figure 8A-B)**. We then utilized the Basso, Beattie, and Bresnahan (BBB) scoring system to assess the recovery of motor function in the rats over an 8-week period following SCI 62. Before injury, all rats exhibited normal motor behavior (BBB score = 21). Immediately after surgery, complete hindlimb paralysis was observed (BBB score = 0), confirming the successful establishment of the SD rats SCI model **(Figure 8C)**. In the AG group, the BBB score reached only 2.8 ± 0.79 after 8 weeks, indicating that without bioactive substances, the hydrogel alone has limited capacity to promote recovery in rats** (Movie S1)**. After 8 weeks, both the PAG group (5.8 ± 1.03) and the PAG-BDNF group (8.6 ± 2.63) showed improvements in BBB scores **(Movies S2 and S3)**. Nevertheless, the PAG-BDNF group demonstrated a markedly enhanced functional recovery as a consequence of the addition of BDNF. At the eighth week, we used footprint analysis to assess hindlimb recovery in the rats. As shown in the **Figure 8D**, rats in the AG group dragged their hindlimbs passively, resulting in wavy footprints. In the PAG group, partial recovery was observed. Although one side still showed intermittent drag marks, the other hindlimb displayed clear footprints with minimal dragging, indicating that the PAG hydrogel facilitated some recovery in SCI animals by enabling the hindlimbs to support body weight. Rats in the PAG-BDNF group exhibited more coordinated hindlimb movements, with clear footprints and no significant dragging, demonstrating that the addition of BDNF enhanced the neural repair capabilities of the PAG hydrogel. Regarding stride width and stride length, PAG-BDNF (63.99 ± 2.7 mm and 183.57 ± 16.24 mm) performed better than AG (111.94 ± 7.37 mm and 0 mm) and PAG (84.33 ± 3.46 mm and 135.79 ± 10.61 mm), approaching the performance of the Sham group (47.85 ± 6.18 mm and 215 ± 13.14 mm)** (Figure 8E-F)**.

The functional recovery may be related to changes in neural structure and pathophysiology. The pathological state of the transected spinal cord was assessed at 8 weeks using gross anatomy and routine magnetic resonance imaging (MRI) 63. The injured spinal cord showed obvious cavities and scar tissue, resulting from the loss of neural tissue. The results of the MRI scans demonstrated a notable reduction in the injury area in both the PAG-BDNF and PAG groups, in comparison to the AG group **(Figure 8H)**. We further examined the structure of the injury site using H&E and Masson staining **(Figure 8I)**. The H&E results demonstrated that the cavity in the AG group was noticeably larger than in the PAG and PAG-BDNF groups, with the cavity in the PAG-BDNF group being notably smaller than in the PAG group. These findings indicate that as the hydrogel enhances tissue repair capabilities, increased nerve fiber regeneration reduces the cavity area post-SCI. Overall, these results demonstrate that the PAG-BDNF hydrogel can promote motor function recovery after SCI.

### The effects of neural regeneration-enhanced triple-network hydrogel implantation on neuroregeneration, differentiation, nerve fiber regeneration, and endogenous NSC recruitment after SCI

**Figure 9:**
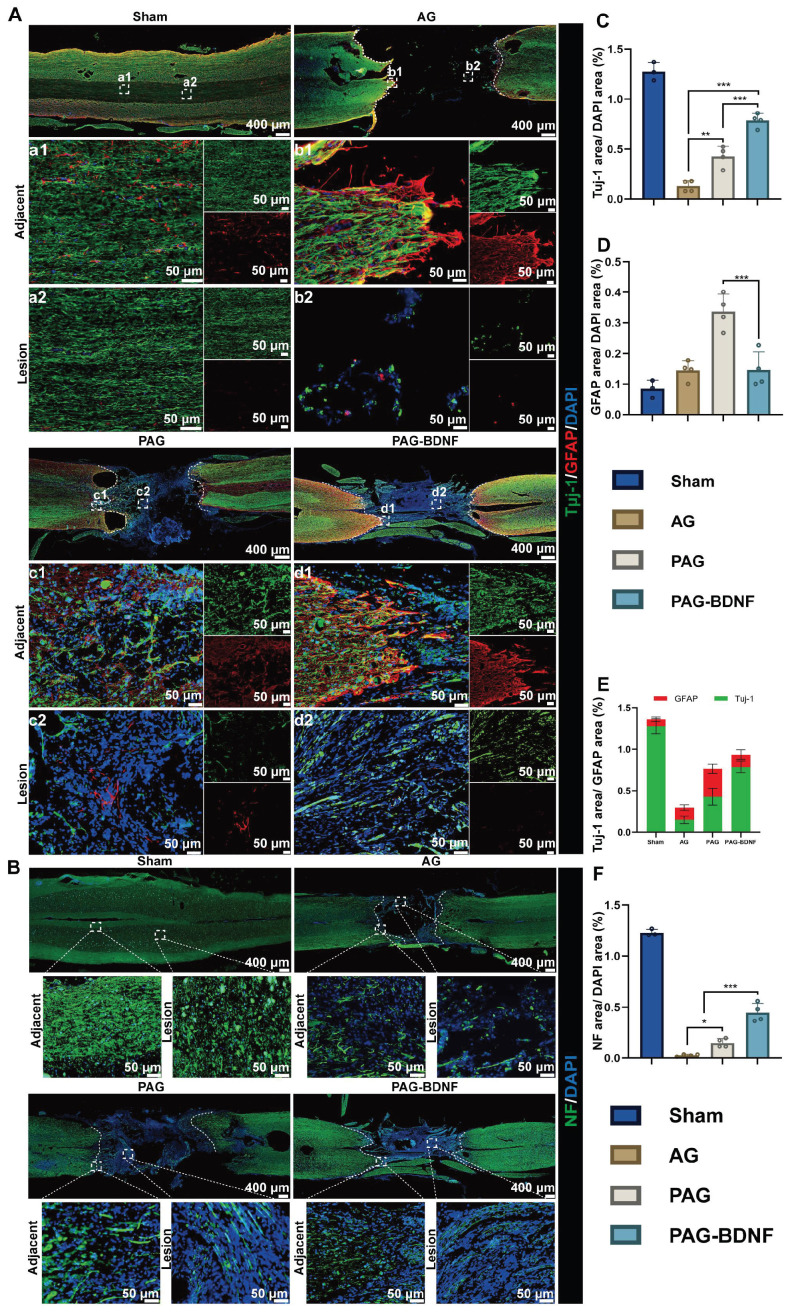
The neural regeneration-enhanced triple-network hydrogel promotes neuroregeneration, differentiation and nerve fiber regeneration. A). Representative tissue sections stained with Tuj-1 (green) and GFAP (red) from different experimental groups at 8 weeks post-SCI are shown. The boxed areas in the gross immunohistofluorescence images, indicating adjacent and lesion regions, are magnified in panels a1-d1 and a2-d2 for detailed observation. B). Representative tissue sections stained for NF (green) from various groups at 8 weeks post-SCI. The boxed areas in the gross immunohistofluorescence images highlight adjacent and lesion regions. C). Quantitative analysis of Tuj-1/GFAP area within the SCI lesion (Sham group n = 3, AG, PAG, PAG-BDNF groups n = 4). D). Quantitative analysis of Tuj-1/DAPI area in the SCI lesion (Sham group n = 3, AG, PAG, PAG-BDNF groups n = 4). E). Quantitative analysis of GFAP/DAPI area in the SCI lesion (Sham group n = 3, AG, PAG, PAG-BDNF groups n = 4). F). Quantitative analysis of NF/DAPI area in the SCI lesion (Sham group n = 3, AG, PAG, PAG-BDNF groups n = 4). Error bars indicate the SD (*P < 0.05, **P < 0.01, ***P < 0.001).

To gain further insight into the effects of PAG-BDNF on functional recovery following SCI, we employed immunofluorescence labelling to detect and evaluate neurogenesis, astrocyte formation and axonal regeneration in the injured area. As illustrated in** Figure 9A and 9C-E**, numerous Tuj-1-positive cells were observed in the lesion site and injured area in the PAG-BDNF and PAG groups, whereas very few Tuj-1-positive cells were detected in the AG group, indicating the presence of newly formed neurons. The formation of glial scars after SCI is one of the key factors inhibiting functional recovery 64. The level of GFAP indicates the formation of glial scars. The results demonstrated that GFAP-positive astrocytes were fewer in the injury area of the PAG-BDNF group compared to the PAG and AG groups. These results suggest that both PAG and PAG-BDNF, due to the presence of PRP, promote neural regeneration more effectively than AG without bioactive substances. However, PRP does not have the capacity to induce the directional differentiation of NSCs into neurons, resulting in a concomitant increase in GFAP within the PAG group. In contrast, PAG-BDNF improved this outcome by reducing the number of astrocytes, decreasing GFAP expression, and increasing the ratio of Tuj-1 expression, thereby creating a more favorable microenvironment for subsequent axonal regeneration. This is attributed to the inclusion of BDNF, which makes the composite triple network hydrogel more beneficial for use in nerve regeneration.

Neurofilament (NF) is a cytoskeletal protein that plays a role in the distribution of nerve fibers and the growth of axons 65-67. We quantified axonal regeneration by examining NF-positive axons regenerating into the lesion area **(Figure 9B, 9F)**. The presence of NF-positive fibers was noted in AG, PAG, and PAG-BDNF groups. However, the AG group exhibited a paucity of NF-positive fibers in the injury area, while the PAG group had some NF-positive fibers, but they appeared disorganized and clustered around the material. In the PAG-BDNF group, the highest number of NF-positive fibers was observed, with the regenerated axons tending to connect to the central injury area. This suggests that the PAG-BDNF hydrogel can induce axonal regeneration at the injury site. In the PAG-BDNF group, the highest number of NF-positive fibers was observed, with the regenerated axons tending to connect to the central injury area. This indicates that the PAG-BDNF hydrogel has the potential to facilitate axonal regeneration at the site of injury. We then used Western blotting (WB) to quantify NF protein expression in the SCI area **(Figure 11C, 11D)**. The quantitative results were in accordance with the immunofluorescence data, indicating that the PAG-BDNF group exhibited superior performance compared to both the PAG and AG groups. Furthermore, neurogenesis encompasses the migration of endogenous NSCs, which are capable of proliferating and differentiating into functional cells (neurons, astrocytes, oligodendrocytes, etc.) when recruited to the injury area 68, 69. In both the PAG-BDNF and PAG groups, the presence of Nestin-positive cells in the injury area was observed, indicating that endogenous NSCs had migrated to the lesion site and undergone further differentiation into new neurons **(Figure 11A)**. In summary, these experimental results demonstrate that PAG-BDNF has exceptional neuroregenerative properties. It promotes neuronal proliferation, axonal growth, and endogenous neurogenesis while simultaneously reducing glial scar formation.

### The implantation of neural regeneration-enhanced triple-network hydrogel promotes angiogenesis

**Figure 10:**
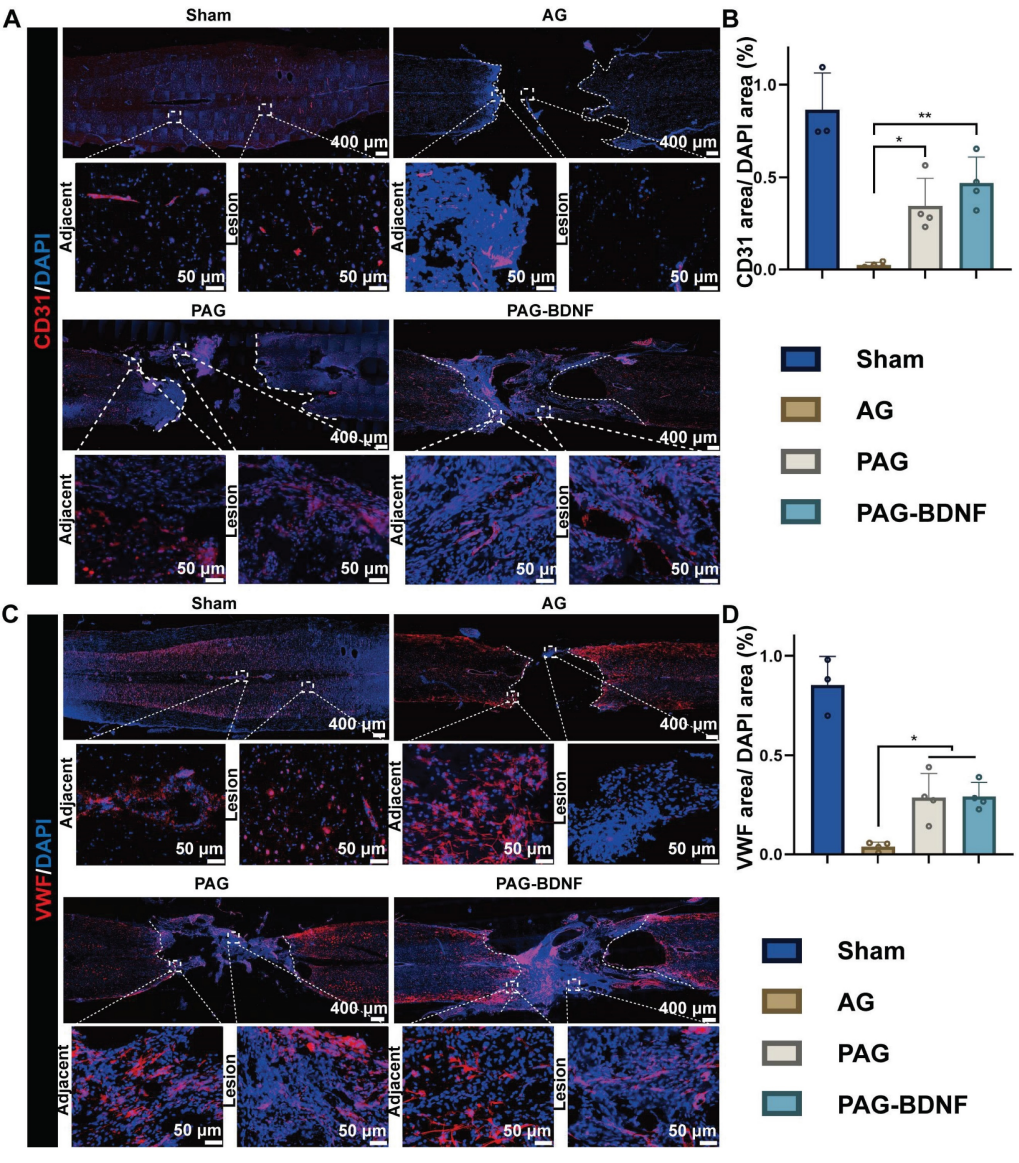
The neural regeneration-enhanced triple-network hydrogel promotes angiogenesis. A). Representative tissue sections stained for CD31 (red) from various groups at 8 weeks post-SCI. The boxed areas in the gross immunohistofluorescence images highlight adjacent and lesion regions. B). Representative tissue sections stained for VWF (red) from various groups at 8 weeks post-SCI. The boxed areas in the gross immunohistofluorescence images highlight adjacent and lesion regions. C). Quantitative analysis of CD31/DAPI area at the SCI lesion site (Sham group n = 3, AG, PAG, PAG-BDNF groups n = 4). D). Quantitative analysis of VWF/DAPI area at the SCI lesion site (Sham group n = 3, AG, PAG, PAG-BDNF groups n = 4). Error bars indicate the SD (*P < 0.05, **P < 0.01).

**Figure 11:**
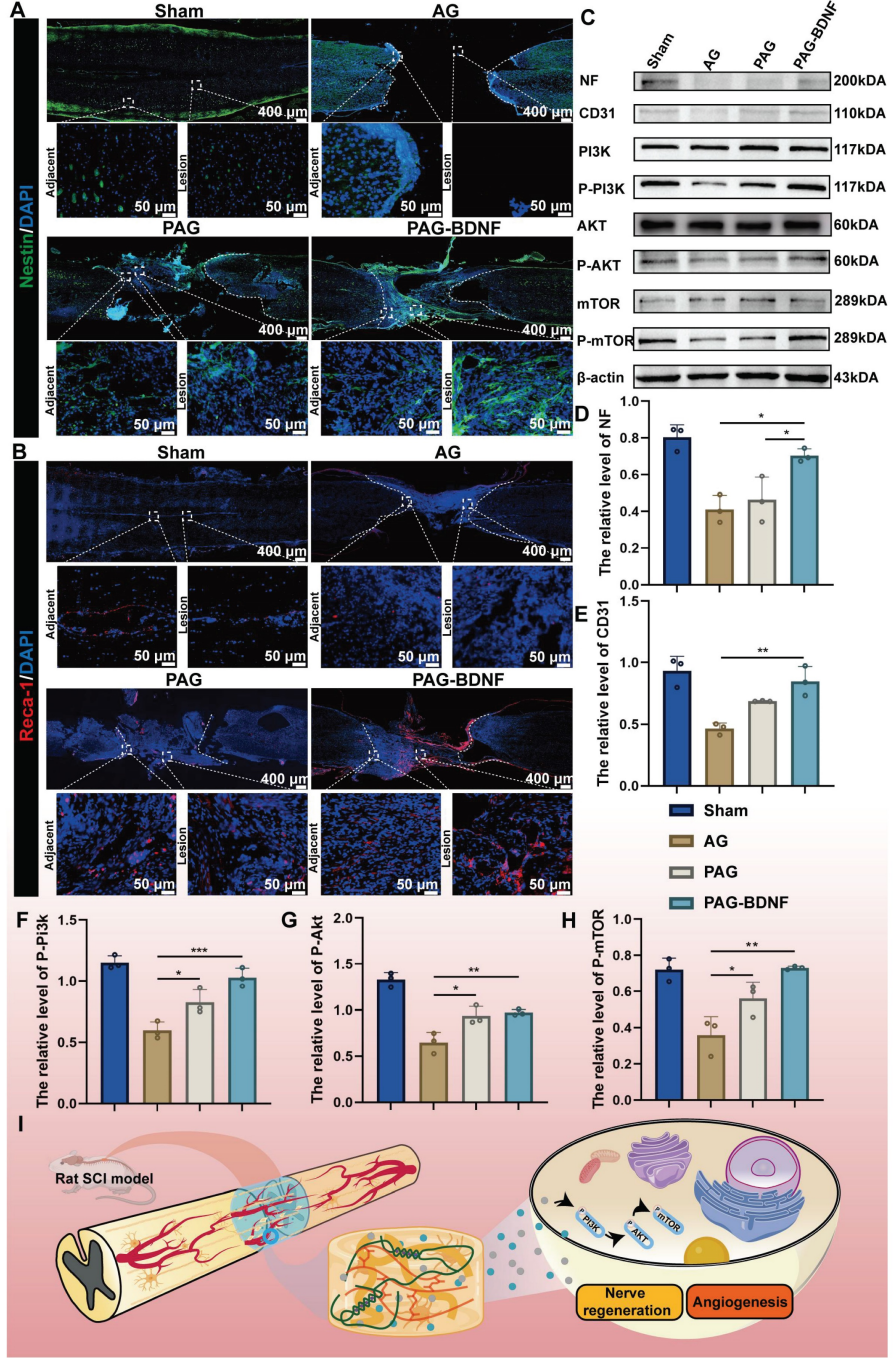
The neural regeneration-enhanced triple-network hydrogel promotes the recruitment of endogenous NSCs and vascular regeneration, contributing to the repair of SCI through its underlying mechanisms. A). Representative images of tissue sections stained for Nestin (green) from different groups, 8 weeks post-SCI. Boxed regions indicate adjacent and lesion areas in each immunohistofluorescence image. B). Representative images of tissue sections stained for Reca-1 (red) from different groups, 8 weeks post-SCI. Boxed regions indicate adjacent and lesion areas in each immunohistofluorescence image. C). Protein expression levels of NF, CD31, and proteins involved in the PI3K/AKT/mTOR pathway. β-actin was used as a loading control. D)-H). Graphs depicting the quantification of protein band intensities (n = 3 per group). Error bars indicate the SD (*P < 0.05, **P < 0.01, ***P < 0.001). I). The neural regeneration-enhancing triple-network PAG-BDNF hydrogel promotes nerve and vascular regeneration via the PI3K/AKT/mTOR pathway.

Following a traumatic SCI, the disruption of the local microvascular system in the affected area initiates a cascade of pathological responses associated with both primary and secondary injuries, leading to an unfavorable microenvironment for cellular regeneration at the injury site 70. PRP gel is abundant in various angiogenic growth factors, including PDGF and VEGF, which stimulate microvascular regeneration 51. The restoration of microvasculature also aids the survival of neural cells in the injury area after SCI 71. Therefore, 8 weeks post-SCI, we monitored vascular regeneration in the lesion area through immunostaining for CD31, von Willebrand factor (VWF), and mouse monoclonal to endothelial cell (Reca-1) 27, 62. Immunohistochemical results from **Figures 10A-B, and 11B** showed significant expression of CD31, VWF, and RECA-1 in the PAG-BDNF and PAG groups compared to the AG group. Quantitative analysis confirmed that CD31 and VWF expression in the PAG-BDNF and PAG groups was significantly higher than in the AG group, with no statistically significant difference between PAG-BDNF and PAG **(Figure 10C, 10D)**. WB analysis of CD31 protein expression corroborated these findings, showing no statistical difference between PAG-BDNF and PAG but a clear advantage over AG **(Figure 11C, 11E)**. In summary, these results suggest that enhanced vascular regeneration is attributable to PRP's superior angiogenic properties, providing a favorable platform for neural repair and reconstruction after SCI.

### Potential molecular mechanisms by which neural regeneration-enhanced triple-network hydrogels promote SCI repair

Our results demonstrate that the triple-network hydrogel significantly impacts both nerve and vascular regeneration, although the precise molecular mechanisms remain unclear. Previous studies have shown that the phosphatidylinositol 3-kinase (PI3K)/protein kinase B (AKT)/mammalian target of rapamycin (mTOR) signaling pathway plays a crucial role in regulating neuronal activity, axonal regeneration, cell proliferation and angiogenesis 4, 33, 72. Of this pathway, PI3K/AKT is well known as a relevant signaling pathway mediating neuronal survival 73. PI3K promotes neurite growth and retraction in neuronal cell lines 73, AKT is closely associated with neuronal regeneration 74, 75, and its downstream molecule mTOR is also responsible for neurons' cellular and physiological regeneration 76. Additionally, this pathway influences angiogenesis and vascular remodeling 77-79. We speculate that PAG-BDNF hydrogel may activate the PI3K/AKT/mTOR signaling pathway, which in turn could lead to repair and regeneration after SCI through the dual pathways of angiogenesis and neural regeneration. To investigate the potential molecular mechanisms by which the hydrogel promotes spinal cord regeneration, total protein was extracted from the regenerated spinal cord of four experimental groups for observation of the expression of phosphorylated proteins of the pathway. As shown in the **Figure 11C, 11F-H and Figure S2-4**, after 8 weeks, the expression of p-PI3K, p-AKT, and p-mTOR was relatively high in the PAG-BDNF and PAG groups, whereas the AG group showed very low expression levels. The proposed potential mechanism through which PAG-BDNF hydrogel promotes nerve and vascular regeneration is through the co-stimulation of the PI3K/AKT/mTOR pathway **(Figure 11I)**.

In the neural-enhancing triple-network hydrogels designed herein, the cocktail effects of slow-release PRP and BDNF synergistically promoted the recruitment, migration, proliferation and differentiation of rat NSCs *in vivo* and *in vitro*. Our study shows that PRP strongly promotes the proliferation of nerve cells, but it has no effect on the directed differentiation of NSCs to neurons, and it similarly promotes the differentiation of NSCs to astrocytes, exacerbating scar formation. The addition of BDNF alleviated the defects of PRP in neural differentiation. Our results showed that, both *in vivo* and *in vitro*, PAG-BDNF reduced astrocyte formation compared to PAG hydrogel while promoting neuronal regeneration, effectively inhibited the regeneration of glial scarring, and protected neuronal growth and axon regeneration, which was beneficial to the reconstruction and recovery of function after spinal cord injury in rats.

Regeneration of blood vessels is also a critical step in the rehabilitation process of spinal cord injury, and the degree of regeneration of the spinal cord is closely related to the degree of regeneration of blood vessels 75. Our study demonstrated that PRP-loaded triple-network hydrogels PAG and PAG-BDNF had a strong ability to promote vascular regeneration both *in vitro* and *in vivo*. It promoted the regeneration of blood vessels in the damaged spinal cord region and improved the blood supply in the SCI region, which is consistent with the results of other studies about revascularization for other disease models 80, 81. Based on the above results, we suggest that the vascular regeneration ability of PRP-loaded triple network hydrogels provides some support for functional reconstruction and recovery in SCI rats.

In this paper, using PRP gel and BDNF growth factor, which are widely used in clinical tissue repair, and two biosafety hydrogel materials, Alg and GelMA, we have designed a non-toxic, biomimetic and beneficial for nerve-regeneration nerve-enhancing triple-network hydrogel using a simple two-step method. Firstly, the triple-network hydrogel was innovatively prepared in this paper. Secondly, the triple-network hydrogel cleverly used Ca^2+^ to combine Alg and PRP, acting as a cross-linking agent for Alg and as an activator for PRP, while GelMA provides support as the third network. Moreover, the hydrogel promotes both nerve regeneration and vascular regeneration, which has more potential than single-function hydrogel products. In addition, the addition of BDNF improves the inadequacy of PRP hydrogel in not enabling the targeted differentiation of NSCs into neurons, expands the possible application of PRP and its derivatives in the field of nerve repair, and provides a new therapeutic option for the rehabilitation of SCI patients. The ease of handling and biosafety of the design make the hydrogel have the potential for translation and application to the clinic.

Although this experiment demonstrates the feasibility of PAG-BDNF, there are some limitations to this experiment. Firstly, although our hydrogel had promising results in the SCI rat model, we need to demonstrate whether it can play the same role within primates. In addition, although we made some exploration of the pathway mechanisms by which PAG-BDNF hydrogels may function, the underlying mechanisms still need to be further investigated.

## Conclusion

In this study, we developed a neural regeneration-enhanced triple-network hydrogel, PAG-BDNF, that facilitates both neural and vascular regeneration for the treatment of traumatic SCI. The PAG-BDNF hydrogel is injected into the injury cavity after crosslinking with calcium ions, followed by UV crosslinking to stabilize the damaged area, facilitating tissue repair. Our experimental results demonstrate that the PAG-BDNF hydrogel promotes the proliferation, adhesion, migration, and tube formation of HUVECs *in vitro*, while also enhancing the proliferation and migration of NSCs, inducing their differentiation into neurons, and inhibiting their differentiation into astrocytes. *In vivo* experiments, including BBB scoring and footprint analysis, reveal that PAG-BDNF improves spinal cord function recovery in rats with SCI. Immunofluorescence data further indicate that PAG-BDNF hydrogel promotes neural regeneration, inhibits glial scar formation, fosters axonal growth, facilitates the migration of endogenous NSCs, and enhances vascular regeneration. More importantly, the PAG-BDNF hydrogel induce neurogenesis and angiogenesis probably by activating the PI3K/AKT/mTOR signaling pathway. In conclusion, the neural regeneration-enhanced triple-network hydrogel we developed has excellent dual biological functions of neural regeneration and angiogenesis, and it is a straightforward, viable, and promising therapeutic strategy for SCI regeneration.

## Materials and Methods

### Preparation of PRP, Alg-GelMA hydrogels, PRP-Alg-GelMA hydrogels, PRP-Alg-GelMA-BDNF hydrogels

Under sterile conditions, PRP was prepared from whole blood using a double-centrifugation method. Blood was collected via cardiac puncture from healthy SD rats and centrifuged at 200 × g for 15 minutes to separate the upper blood layer, containing leukocytes, platelets and plasma. This fraction was transferred to a new 15 mL centrifuge tube for a second centrifugation at 400 × g for 20 minutes. The liquid again separated into layers: platelet-poor plasma (PPP) in the upper layer and platelet-rich precipitate in the lower layer. Approximately three-quarters of the upper PPP was discarded, leaving just enough plasma to resuspend the platelet pellet, resulting in PRP.

Alg was obtained from Tianjin Guangfu Technology Development Co., Ltd. (9005-38-3, Tianjin, China, Molecular weight=216.12 kDa, Purity: CP). GelMA (EFL-GM-60, Suzhou, China 82) was purchased from Yongqinquan Intelligent Equipment Co., Ltd. (Suzhou, China). The preparation of Alg-GelMA (AG) hydrogel involves dissolving Alg (0.5%, 1%, 2% w/v), GelMA (5% w/v), and lithium phenyl-2,4,6-trimethylbenzoylphosphinate (LAP) (0.25% w/v) in PBS. Alg is first crosslinked by adding CaCl_2_ (2% w/v) dropwise, followed by 20 seconds 405nm UV exposure to form the GelMA hydrogel.

The preparation of PRP-Alg-GelMA (PAG) hydrogel involves dissolving Alg (1% w/v), GelMA (5% w/v), and LAP (0.25% w/v) in PBS containing varying concentrations of PRP (5%, 10%, 20% v/v). Alg and PRP are first crosslinked into a dual-network gel by adding CaCl_2_ (2% w/v) dropwise, followed by 20 seconds 405nm UV exposure to form the GelMA hydrogel.

The preparation of PRP-Alg-GelMA-BDNF (PAG-BDNF) hydrogel involves dissolving Alg (1% w/v), GelMA (5% w/v), LAP (0.25% w/v), and BDNF (200 ng/mL) in PBS containing 20% v/v PRP. The Alg and PRP are first crosslinked into a dual-network gel by dropwise addition of CaCl_2_ (2% w/v), followed by 20 seconds 405nm UV exposure to crosslink the GelMA into a third network.

### Characterization of Alg-GelMA hydrogels, PRP-Alg-GelMA hydrogels, PRP-Alg-GelMA-BDNF hydrogels

The storage modulus (G') and loss modulus (G") of the hydrogels were measured at room temperature using a rheometer (DHR-2, TA Instruments, USA) equipped with a parallel plate fixture. The upper fixture had a diameter of 25 mm, with the gap between the sample and fixture set to 1 mm. At room temperature, the changes in G' and G" were recorded at a fixed frequency of 1 Hz and 1% strain. Additionally, the oscillatory strain was held constant at 1% while the angular frequency was varied from 0.1 rad/s to 100 rad/s. For viscosity characterization, the hydrogels were tested at shear rates ranging from 0.1 s⁻¹ to 100 s⁻¹. For the characterization of self-healing, the oscillation frequency was controlled to be 1 Hz and cycled at 2% and 200% of the oscillatory strain.

The microstructure of the hydrogel was examined using a cold-field emission scanning electron microscope (SU8020, Hitachi). The samples were lyophilized after treatment, and a gold layer was sputter-coated onto the freeze-dried hydrogel prior to examination.

The initial mass (W1) of the hydrogel sample (500 µL) was weighed, and the sample was then soaked in PBS at room temperature. At predetermined time intervals (1 h, 3 h, 5 h, 8 h, 12 h, 24 h, 48 h), the PBS on the surface of each hydrogel was removed using filter paper, and the swollen hydrogel was weighed (W2). The mass swelling ratio was calculated using the following equation: Mass Swelling Ratio (%) = (W2 - W1) / W1 × 100%.

The degradation performance of the material was evaluated through a degradation experiment. The samples of different groups were immersed in PBS at 37°C and weighed after complete swelling (W1). At predetermined time points, the samples were removed, and excess water was blotted away before weighing the samples again (Wt). The degradation rate was determined using the following formula: Degradation Rate (%) = (W1 - Wt) / W1 × 100%.

The GFs release was quantified using a BCA Protein Assay Kit. A 200 µL sample of PAG-BDNF hydrogel and an equal volume of PRP-BDNF gel were placed separately into 2 mL of sterile PBS. Protein concentration was measured at different time points by taking samples from the PBS solution.

### Extraction and identification of neural stem cells

Neural stem cells (NSCs) were isolated from the hippocampus of embryonic SD rats. Briefly, under sterile conditions, the collected hippocampal tissue was digested into a single-cell suspension using Accutase solution (Sigma, USA), and tissue debris was filtered using a 70μm cell strainer (Biosharp, China). The cells were then seeded into T25 culture flasks (BIOFIL, TCF012050) and cultured in DMEM/F12 medium (GIBCO, USA) supplemented with 20ng/ml EGF (PeproTech, USA), 20ng/ml bFGF (TargetMol, USA), 2% B27 (GIBCO, USA), and 1% penicillin-streptomycin (GIBCO, 15140122). The NSCs were cultured at 37°C in 5% CO_2_, and after forming neurospheres, they were passaged every 3-4 days. In this study, 2nd to 4th generation NSCs were used. NSCs were identified through immunofluorescence staining. The cells were fixed with 4% paraformaldehyde for 30 minutes and washed three times with PBS. They were then blocked with 10% goat serum (Sigma, USA) for 30 minutes. After blocking, the cells were incubated with the primary antibody (Nestin 1:600, ab196908, Abcam, U.K.) at 4°C for 24 hours. Following incubation, the cells were washed three times with PBS and incubated with the secondary antibody (Alexa-Fluor 488, 1:600, Invitrogen, USA) for 40 minutes. Nuclear staining was performed with DAPI for 30 minutes. The staining results were visualized using a laser confocal microscope (FV3000, Olympus, Japan).

### Biocompatibility of hydrogels *in vitro*

The experimental groups included the AG group, the PAG group, and the PAG-BDNF group. Live/dead staining of human umbilical vein endothelial cells (HUVECs) and NSCs after co-culture with the materials was used to assess cytotoxicity. This was achieved using Calcein-AM/Propidium Iodide (Calcein-AM/PI) (EFL, China) staining, which was observed under a fluorescence microscope (Olympus Corporation, Japan). The Cell Counting Kit-8 (CCK-8) (DOJINDO, Japan) assay was employed to assess cell proliferation and viability. HUVECs and NSCs were co-cultured with hydrogels in 24-well plates, and at various time points, 1% CCK-8 solution was added to the wells. After a 2-hour incubation, 100 µL of the supernatant was transferred to a 96-well plate, and the optical density (OD) was measured at 450 nm using a microplate reader (BioTek). Quantitative analysis was conducted with ImageJ software (NIH, Bethesda, MD).

### Cytoskeletal staining

The cell morphology on the material surface was examined using DAPI and FITC-Phalloidin staining. HUVECs were seeded onto 24-well plates coated with hydrogels. After 1 day of co-culture, the cells were fixed in 4% paraformaldehyde for 30 minutes, followed by three PBS washes to eliminate any remaining paraformaldehyde. The cells were then permeabilized with 0.5% Triton X-100 for 30 minutes. Actin filaments were stained using FITC-Phalloidin (Biosharp, China), and cell nuclei were labeled with DAPI (Solarbio, China). The cytoskeleton was observed using a fluorescence microscope (Olympus Corporation, Japan).

### Wound healing assay

First, draw positioning lines at the bottom of a six-well plate using a marker. Then, seed HUVECs in the six-well plate until they reach 90% confluence. Use the tip of a 1000µL pipette to scrape a vertical line through the monolayer of cells. After co-culturing with the hydrogel for 12, 24, and 48 hours, observe and record the wound width under a microscope (Olympus Corporation, Japan). ImageJ software is used to measure the distance between the edges of the scratch.

### Cell invasion assay

Following 24 hours of serum-free incubation, introduce 100μL of NSCs or HUVECs cell suspension into the upper chamber of the transwell. After another 24-hour incubation, fix the cells with 4% paraformaldehyde for 15 minutes and rinse three times with PBS. Remove any remaining cells in the upper chamber using a cotton swab. Stain the cells with 0.1% crystal violet for 20 minutes, then observe them under an inverted microscope (Olympus Corporation, Japan). Use ImageJ software to analyze the results.

### Tubule formation assay

Add 50μL of Matrigel to a 96-well plate, and once the Matrigel has solidified, resuspend HUVECs in cell/hydrogel extracts. Inoculate the cells onto the Matrigel surface and incubate for 8 hours. After incubation, evaluate network formation under a bright-field microscope. Capture images using an inverted microscope (Olympus Corporation, Japan) and perform quantitative analysis with ImageJ software.

### Differentiation of NSCs in hydrogels

Immunofluorescence labeling was employed to assess the differentiation of NSCs in various hydrogels. As previously described, the NSC differentiation experiment was divided into four groups: Control (PBS), AG, PAG, and PAG-BDNF. NSCs from each group were cultured for 7 days and labeled with the Tuj-1 antibody (1:1000, Abcam, U.K.) to examine NSC differentiation and neurite outgrowth. For assessing NSC differentiation into astrocytes, another group of NSCs, cultured in different hydrogels, was labeled with the GFAP antibody (1:600, CST, USA). The immunofluorescence staining procedure followed the same method used for NSC identification.

### Quantitative polymerase chain reaction assays

Total RNA was isolated from cells using TRIzol reagent (Sigma, China). 2 ug of RNA were reverse-transcribed into cDNA using the cDNA reverse transcription kit (Thermo Fisher Scientific, USA). Quantitative polymerase chain reaction (qPCR) was carried out with a SYBR Green PCR kit (Roche, Shanghai, China) on a CFX96 real-time PCR detection system (Bio-Rad Laboratories, Inc.). The amplification protocol consisted of pre-denaturation at 94°C for 2 minutes, followed by 35 cycles of denaturation at 94°C for 10 seconds, annealing at 60°C for 15 seconds, and extension at 72°C for 30 seconds, with a final extension at 72°C for 5 minutes. Primers were synthesized by Sangon Biotech (China), and the sequences are listed in **Table S1**. GAPDH was used as the reference gene. The 2^-ΔΔCt^ method was used for analysis.

### Construction of an animal model of SCI

The animal experiments were conducted in accordance with the ARRIVE guidelines and adhered to the ethical standards established by the Institutional Animal Ethics Committee of Jilin University (permission number SY202409015). Adult female SD rats (weight, 200-250g, n = 27) were anesthetized and fixed on the table. In order to adequately expose the spinal cord at T9-10, a laminectomy was performed at the T9 level after the dorsal surfaces were shaved and sterilized with 75% alcohol. Spinal cord tissue was removed using ophthalmic scissors, hemostasis was achieved with gauze, and the area was rinsed with saline. Ca^2+^-crosslinked AG, PAG, and PAG-BDNF hydrogels were then injected into the lesion site. The defect was exposed to UV light for 20 seconds after the hydrogel had completely filled the defect. The skin was closed in layers and the soft tissues were sutured. Until the rats regained normal urination, the bladder was emptied at least twice a day.

### *In vivo* magnetic resonance imaging

Rats were anaesthetised and restrained in the supine position using a 3.0 T MRI scanner (Philips Ingenia CX Magnetic resonance, Netherlands) 8 weeks after surgery. Conventional MRI was used to obtain sagittal T2-weighted images.

### Functional recovery and analysis of the footprint

The Basso-Beattie-Bresnahan (BBB) locomotor rating scale and footprint analysis were used to assess the recovery of hind limb motor function in rats on a weekly basis. Rats were placed in an open field where they could run around freely. All rats were scored weekly for 4 minutes by two independent observers blinded to treatment group. To analyze the footprints, the rats' hind paws were stained with blue ink and the rats were then allowed to walk in a straight line on white paper. Stride length and width were then measured and analyzed.

### Histological staining and immunofluorescence staining

After 8 weeks, the rats were sacrificed. Intracardiac perfusion with isotonic saline and 4% paraformaldehyde followed. Spinal cord tissue was fixed in 4% paraformaldehyde for 24 hours. It was then dehydrated in 30% sucrose for 24 hours. The processed spinal cord tissue was cryosectioned at 15μm thickness, stained with H&E and Masson's stain, and examined by light microscopy. The method for immunofluorescence staining is the following. The immunofluorescence primary antibodies-Tuj-1 (1:1000, ab18207, abcam, U.K.), GFAP (1:600, #3670, CST, USA), NF200 (1:300, #30564, CST, USA), Nestin (1:100, ab6142, abcam, USA), CD31 (1:100, ab222783, abcam, U.K.), VWF (1:500, GB11020-100, Servicebio, China), and RECA-1 (1:100, ab9774, abcam, U.K.)-were applied to slides and incubated for 24 hours at 4°C. The slides were then washed with PBS. After incubation for 40 min at room temperature away from light, the immunofluorescent secondary antibody (Alexa Fluor 488, 1:600; Alexa Fluor 594, 1:800; Invitrogen, USA) and free antibody were removed by washing with PBS. Finally, DAPI staining reagent were added to the sections, which were then glycerol-sealed and subjected to fluorescence microscopy.

### Western blot analysis

NP40 lysis buffer (Beyotime Biotechnology, Shanghai, China) was added to the tissue, followed by homogenization for 10 minutes. Then centrifuge at 12,000 rpm for 15 minutes at 4°C. To extract total protein from the injured spinal cord, the supernatant was collected. A BCA protein assay kit (Thermo Fisher Scientific, USA) was used to measure total protein concentration. Protein samples (60 μg) were separated by 12% sodium dodecyl sulfate-polyacrylamide gel electrophoresis (SDS-PAGE) and transferred to a polyvinylidene fluoride (PVDF) membrane (0.22 μm) (Millipore, Germany). The membrane was incubated with primary antibodies overnight at 4°C after blocking with 5% non-fat milk for 2 hours at room temperature. (Primary antibodies: anti-NF200 (CST, #30564, USA), anti-CD31 (Abcam, ab222783, U.K.), anti-PI3K (Bioss, 2067R, China), anti-phospho-PI3K (Bioss, 5570R, China), anti-AKT (CST, 9272S, USA), anti-phospho-AKT (CST, 4060S, USA), anti-mTOR (CST, #2983, USA), and anti-phospho-mTOR (CST, 5536S, USA)). The membrane was then incubated with secondary antibody IgG for 1 hour at room temperature after four washes (15 minutes each) with TBST buffer. Protein bands were detected by enhanced chemiluminescence (ECL) after four washes with TBST buffer. Finally, the immunoreactive bands were quantified using ImageJ and GraphPad Prism software.

### Statistical analysis

GraphPad Prism (v9.5.0) software was used for all statistical analysis. At least three samples were included in each group, and results were expressed as mean plus standard deviation. Differences between multiple experimental groups were examined by one-way analysis of variance (ANOVA) and Tukey's multiple comparison test. “ns” means non-significant differences, *P* < 0.05 was considered to indicate statistical significance.

## Supplementary Material

Supplementary figures and table, movie legends.

Supplementary movie 1.

Supplementary movie 2.

Supplementary movie 3.
