# Supplementary material for: Neural-enhancing PRP/Alg/GelMA triple-network hydrogel for neurogenesis and angiogenesis after spinal cord injury via PI3K/AKT/mTOR signaling pathway
Source: Theranostics. 2025 Mar 3;15(9):3837–61. doi: 10.7150/thno.109091 (PMC11980671; doi:10.7150/thno.109091)
Supplement: Supplementary file 1 — Supplementary figures and table, movie legends. [file thnov15p3837s1.pdf]

Title: Neural-enhancing PRP/Alg/GelMA triple-network hydrogel for neurogenesis and angiogenesis after spinal cord injury via PI3K/AKT/mTOR signaling pathway

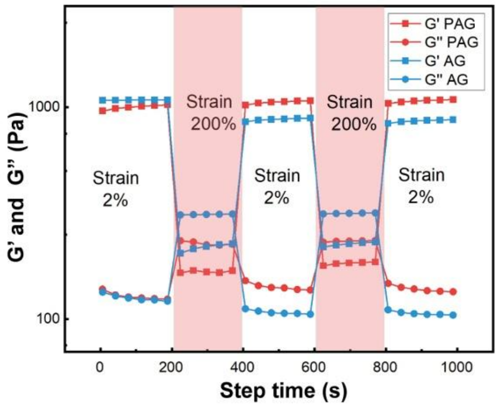

Figure S1. Storage (G′) and loss (G″) moduli of AG and PAG hydrogels after Ca^{2+} and UV crosslinked under varying recycled strain.

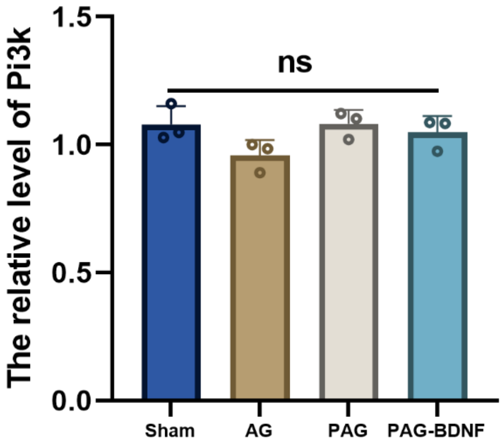

Figure S2. Quantification of Western blot data of PI3K.

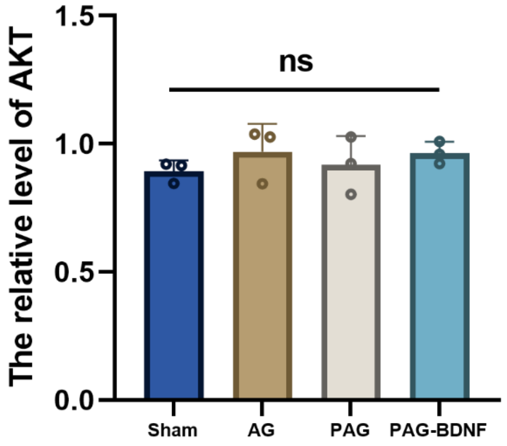

Figure S3. Quantification of Western blot data of AKT.

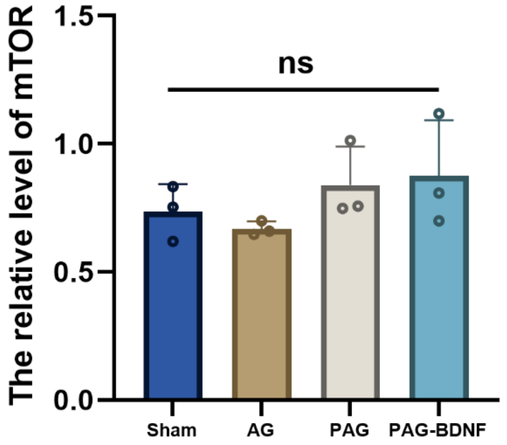

Figure S4. Quantification of Western blot data of mTOR.

Table S1. Primer Sequences

Gene	Forward Primer	Reverse Primer
Human CD31	TGACGGACAGACAGACAGACAC	GGGTCAGGTTCTTCCCATTT
Human VEGF	CCTTGCCTTGCTGCTCTACCTC	GATGATTCTGCCCTCCTCCTTCTG
Rat Tuj-1	TGGACCTGGAGCCTGGAACC	GCCCTCTGTATAGTGCCCTTTGG
Rat GFAP	GAAGGTTGAGTCGCTGGAGGAG	GAAGGTTGAGTCGCTGGAGGAG
Human GAPDH	CACCCACTCCTCCACCTTTGAC	GTCCACCACCCTGTTGCTGTAG
Rat GAPDH	CAAGTTCAACGGCACAGTCAAGG	ACATACTCAGCACCAGCATCACC

Movie S1. Locomotor recovery of the rat after 8 weeks of Alg-GelMA group.

Movie S2. Locomotor recovery of the rat after 8 weeks of PRP-Alg-GelMA group.

Movie S3. Locomotor recovery of the rat after 8 weeks of PRP-Alg-GelMA-BDNF group.
